# Investigating the mechanism of Fuzheng Sanjie Formula in regulating osimertinib resistance in NSCLC via the CAFs-TIMP3-MMP9 axis: a study integrating network pharmacology, data mining

**DOI:** 10.3389/fonc.2026.1851294

**Published:** 2026-08-04

**Authors:** Yifan Wang, Qin Peng, Yuanna Zhang, Haozhu Wang, Jingwen Zhu, Jihong Zhou

**Affiliations:** 1 The Seventh Clinical College of Guangzhou University of Chinese Medicine, Shenzhen, China; 2 Department of Pulmonary Diseases, Shenzhen Bao’an Chinese Medicine Hospital, Shenzhen, China; 3 The Fourth Clinical College of Guangzhou University of Chinese Medicine, Shenzhen, China; 4 Department of Encephalopathy and Psychology, Shenzhen Traditional Chinese Medicine Hospital, Shenzhen, China

**Keywords:** cancer−associated fibroblasts, Fuzheng Sanjie Formula, MMP9, non−small cell lung cancer, Osimertinib resistance, TIMP3

## Abstract

**Background:**

Osimertinib resistance limits EGFR-mutant non-small cell lung cancer (NSCLC) treatment efficacy. Cancer-associated fibroblasts (CAFs), tissue inhibitor of metalloproteinases 3 (TIMP3), and matrix metalloproteinase 9 (MMP9) are involved in resistance. Fuzheng Sanjie Formula (FZSJF) is used clinically, but its mechanism is unclear.

**Objective:**

To explore the mechanism of FZSJF in reversing osimertinib resistance via the CAFs-TIMP3-MMP9 axis using network pharmacology, *in vitro*, and *in vivo* studies.

**Methods:**

The main components of FZSJF were analyzed. Network pharmacology and molecular docking were used to assess binding to MMP9/TIMP3. TCGA data were used for expression and prognostic analysis. CompuSyn was used to calculate the combination index (CI) to assess whether the combination of osimertinib and FZSJF was synergistic Osimertinib-resistant cells (H1975OR) and CAFs were established and treated with control, osimertinib, FZSJF-containing serum, or their combination. Proliferation, invasion, migration, and MMP9/TIMP3 expression were evaluated by CCK-8, Transwell, wound healing, colony formation, Western blot (WB), quantitative real-time PCR (qPCR), and immunofluorescence (IF). TIMP3-silencing CAFs were co-cultured with H1975N cells to assess invasion and MMP9 expression. Nude mouse xenografts (H1975N, H1975OR, H1975N+CAFs) were used to evaluate tumor growth and protein expression.

**Results:**

MMP9 was identified as a key target. FZSJF components and osimertinib showed good binding to MMP9/TIMP3. TCGA revealed high MMP9 and low TIMP3 in lung cancer, with MMP9 linked to shorter overall survival (OS) and TIMP3 to longer OS. CI values confirmed synergy between osimertinib and FZSJF. H1975OR cells and CAFs showed upregulated MMP9 and downregulated TIMP3. FZSJF-containing serum inhibited the proliferation, invasion, and migration of H1975OR cells, upregulated TIMP3, and downregulated MMP9; when combined with osimertinib, these effects were enhanced. TIMP3-silencing CAFs promoted H1975N invasion and MMP9 expression. *In vivo*, H1975OR and H1975N+CAFs tumors grew faster with higher MMP9 and lower TIMP3. FZSJF alone or combined inhibited tumor growth, restored MMP9/TIMP3 balance, without affecting body weight.

**Conclusion:**

FZSJF reverses osimertinib resistance by upregulating TIMP3 and inhibiting CAFs-mediated MMP9 overexpression, restoring MMP9/TIMP3 balance and suppressing NSCLC invasion. This study provides experimental evidence for the efficacy of FZSJF in treating resistant NSCLC.

## 1 Introduction

Lung cancer, also known as primary bronchogenic carcinoma, refers to a malignant tumor originating from the bronchial mucosa or glands of the lungs (1). According to the global cancer statistics report released by the World Health Organization (WHO), there were 2.5 million new cases of lung cancer worldwide in 2022, accounting for approximately one-eighth of all new malignant tumors, and 1.82 million deaths, representing about one-fifth of all cancer deaths. NSCLC is the most predominant histologic type, constituting approximately 80% of all lung cancer patients (2). Although osimertinib has become the standard first-line and second-line treatment for EGFR-mutant non-small cell lung cancer (NSCLC), its efficacy is inevitably limited by acquired resistance. The FLAURA study showed that most patients still experience disease progression within approximately 18–20 months after initiating osimertinib treatment (3), and the literature indicates that the mechanisms of osimertinib resistance are highly heterogeneous. Moreover, the mechanisms underlying osimertinib resistance are very complex. A large number of studies have shown that approximately 30–50% of osimertinib resistance mechanisms remain unexplained (4), suggesting that our understanding of osimertinib resistance is still incomplete, and that there are still undiscovered or insufficiently studied pathways, molecular events, and tumor microenvironment factors that influence osimertinib resistance (5).

Cancer-associated fibroblasts (CAFs) are the most abundant stromal cell type in the NSCLC tumor microenvironment. Studies have shown that CAFs can secrete large amounts of MMP9. Moreover, multiple clinical studies have confirmed that high MMP9 expression is significantly associated with adverse pathological features (such as advanced stage and lymph node metastasis) and poor prognosis in NSCLC and various other malignancies (6, 7). Tissue inhibitors of metalloproteinases (TIMPs) are endogenous inhibitors of the MMP family (8). The loss of TIMP3 directly abolishes its inhibitory effect on MMP9 (9). Simultaneously, when TIMP3 is absent, MMP activity increases, leading to enhanced release of growth factors such as TGF-β, which further induces the transformation of normal fibroblasts into activated cancer-associated fibroblasts (CAFs). This CAF-driven “TIMP3-MMP9 homeostatic imbalance” results in abnormally high and persistently activated MMP9 expression in the tumor microenvironment (10, 11). Moreover, TIMP3 also restricts excessive degradation and disordered crosslinking of the extracellular matrix (ECM) by inhibiting MMPs. In a CAF environment with low TIMP3 expression, the matrix undergoes extensive remodeling, thereby elevating tumor interstitial pressure. This not only promotes mechanical migration of cancer cells but also hinders the penetration of targeted drugs and chemotherapeutic agents (12). Therefore, dysregulation of the CAFs-TIMP3-MMP9 axis may represent an important mechanism underlying osimertinib resistance in NSCLC.

Numerous clinical practices have demonstrated that traditional Chinese medicine (TCM) offers advantages such as efficacy, low toxicity, and individualization in the treatment of osimertinib-resistant NSCLC. The combination of TCM and Western medicine can significantly stabilize disease progression and improve the quality of life for NSCLC patients (13, 14). Professor Zhou Jihong from Guangzhou University of Chinese Medicine, based on the concept that “when healthy qi exists inside, pathogenic factors cannot interfere” and drawing on years of clinical experience, proposed the therapeutic principle of “reinforcing healthy qi and resolving masses” for osimertinib-resistant NSCLC. Accordingly, she developed FZSJF to reinforce healthy qi, tonify deficiency, resolve masses, and reduce swelling, thereby treating NSCLC. However, the mechanism by which FZSJF reverses resistance remains unclear. Therefore, this study aims to further elucidate the mechanism of FZSJF against osimertinib-resistant NSCLC by integrating data mining, cellular experiments, and animal experiments, with the hypothesis that its mechanism may involve regulation of the TIMP3-MMP9 axis, thereby providing a basis for its clinical application.

## 2 Materials

### 2.1 Cells and experimental animals

The NCI-H1975 non-small cell lung cancer cell line was used. Human lung fibroblasts HFL1 were used as normal cancer-associated fibroblasts (NAFs), and HFL1 cells induced by TGFβ were used as CAFs. All cell lines were purchased from Wuhan Pricella Life Science & Technology Co., Ltd. (Wuhan, China).

For experimental animals, 42 male SPF BALB/c nude mice (4 weeks old) and 36 male SPF SD/Wistar rats (7 weeks old) were purchased from Guangdong Weitong Lihua Experimental Animal Technology Co., Ltd. (Guangdong, China) [License No.: SCXK (Yue) 2022-0063]. The animals were housed in the SPF animal facility of Shenzhen BayONE Life Science Technology Co., Ltd. (Shenzhen, Guangdong, China). All animal experiments were approved by the Institutional Animal Care and Use Committee of Shenzhen BayONE Life Science Technology Co., Ltd. (Ethics Approval No.: B0-IRB-20251027-GZZYYDXFSBAQZYY-ZJH-A0028).

### 2.2 Drugs

FZSJF was composed of the following crude herbs: *Fici Hirtae Radix* (20 g), *Scutellariae Radix* (15 g), *Pseudostellariae Radix* (20 g), *Poria* (20 g), *Atractylodis Macrocephalae Rhizoma* (10 g), *Polygoni Cuspidati Rhizoma* (10 g), *Lobeliae Chinensis Herba* (10 g), *Ostreae Concha* (20 g), *Cremastrae Pseudobulbus Pleiones Pseudobulbus* (10 g), *Curcumae Rhizoma* (10 g), *Platycodonis Radix* (10 g), *Hedyotidis Diffusae Herba* (15 g), and *Ranunculi Ternati Radix* (15 g). All herbal pieces were provided by Kangmei Pharmacy of Shenzhen Bao’an Traditional Chinese Medicine Hospital (Shenzhen, China). The herbal pieces were soaked in purified water for 30 minutes, decocted twice using conventional methods, and the combined decoctions were concentrated over low heat, then further concentrated by rotary evaporation, freeze-dried, aliquoted, and stored at 4 °C. Osimertinib powder was purchased from Selleck Biotechnology Co., Ltd. (USA) (Catalog No.: S7297).

### 2.3 Experimental reagents

RPMI Medium 1640 (Cat. No. C11875500CP), penicillin-streptomycin (Cat. No. 15140122), and trypsin (Cat. No. 25200072) were purchased from Thermo Fisher Scientific (USA). Phosphate-buffered saline (PBS, Cat. No. G4202) was purchased from Servicebio (China). The fibroblast culture system (Cat. No. PriMed-iCell-026) was purchased from Shanghai iCell Bioscience Co., Ltd. (China). Fetal bovine serum (FBS, Cat. No. 164210-50) was purchased from Wuhan Pricella Life Science & Technology Co., Ltd. (China). Serum-free cell cryopreservation medium (Cat. No. C40100) was purchased from Suzhou New Life Science & Technology Co., Ltd. (China). The fast RNA extraction kit (Cat. No. AG21023), Evo M-MLV reverse transcription premix kit (Cat. No. AG11728), SYBR^®^ Green Pro Taq HS premix qPCR kit (Cat. No. AG11701), and qPCR primers were purchased from Hunon Accurate Biotechnology Co., Ltd. (China). The BCA protein assay kit (Cat. No. A55864) was purchased from Thermo Fisher Scientific (USA). The fast protein gel kit (Cat. No. 241201) was purchased from Shenzhen Dakewei Biotechnology Co., Ltd. (China). SDS-PAGE sample loading buffer (Cat. No. AR1112-10) was purchased from Wuhan Boster Biological Technology Co., Ltd. (China). Non-fat dry milk (Cat. No. P0216) was purchased from Shanghai Beyotime Biotechnology Co., Ltd. (China). Anhydrous ethanol (Cat. No. E809056) was purchased from Shanghai Macklin Biochemical Technology Co., Ltd. (China). The three-color prestained protein marker (Cat. No. WJ103) was purchased from Shanghai Yeasen Biotechnology Co., Ltd. (China). Rabbit anti-MMP9 (Cat. No. 13667S), rabbit anti-TIMP3 (Cat. No. 5673S), Rabbit anti-FAP (Cat. No. 52818T) and rabbit secondary antibody (Cat. No. 7074S) were purchased from Cell Signaling Technology (USA). Rabbit anti-TIMP3 (Cat. No. ab39184) and Rabbit anti-α-SMA (Cat. No. ab32575) were purchased from Abcam (UK). Mouse anti-GAPDH (Cat. No. 10494-1-AP) and rabbit anti-TIMP3 (Cat. No. 10858-1-AP) were purchased from Proteintech Group, Inc. (Wuhan, China). Mouse anti-MMP9 (Cat. No. GB150096-50), DAPI staining reagent (Cat. No. G1012), anti-fluorescence quenching mounting medium (Cat. No. G1401), Triton X-100 (Cat. No. GC204003), TBST (Cat. No. G2150), PBST (Cat. No. G2157), antibody diluent (Cat. No. G2025), electrophoresis buffer powder (Cat. No. G2081), transfer buffer powder (Cat. No. G2017), Silencing lentivirus packaging (Cat. No. GC1049) and bovine serum albumin (BSA, Cat. No. GC305010) were purchased from Servicebio (China). Membrane stripping buffer (Cat. No. P1650) was purchased from Beijing Applygen Co., Ltd. (China). PEG300 (HY-Y0873), Tween 80 (HY-Y1891), and dimethyl sulfoxide (DMSO, Cat. No. HY-Y0320C) were purchased from MedChemExpress (USA). The ECL Western blotting chemiluminescent substrate (Cat. No. 180-5001) was purchased from Shanghai Tanon Life Science Co., Ltd. (China). Crystal violet staining solution (Cat. No. G1062) was purchased from Beijing Solarbio Science & Technology Co., Ltd. (China). The CCK-8 working solution (Cat. No. GK10001) was purchased from Shanghai Hongye Biotechnology Co., Ltd. (China). Matrigel basement membrane matrix (Cat. No. R32345) was purchased from Shanghai Yuanye Biotechnology Co., Ltd. (China). 4% paraformaldehyde fixative (Cat. No. BL539A) was purchased from Bolaiyuan (Tianjin) Biotechnology Co., Ltd. (China). Ready-to-use tribromoethanol solution (Cat. Nos. M2920 and M2820) was purchased from Nanjing Aibe Biotechnology Co., Ltd. (China). Normal saline was purchased from Sichuan Kelun Pharmaceutical Co., Ltd. (Cat. No. L25081213, China).

## 3 Methods

### 3.1 Network pharmacology, molecular docking, and data mining

#### 3.1.1 Network pharmacology and molecular docking

The active components and corresponding targets of FZSJF were identified using UPLC-Q-TOF-MS/MS combined with the TCMSP and BATMAN-TCM databases and relevant literature. Overlapping targets among NSCLC, cancer-associated fibroblasts (CAFs), and osimertinib resistance were screened using the GeneCard, OMIM, TTD, and DrugBank databases. All target names were standardized using the UniProt database. A Venn diagram was then generated using the ggVennDiagram package in R 4.4.2 to obtain the intersecting targets between the formula and the diseases. A “herb-component-disease-target” visualization network was constructed using Cytoscape v3.7.2. A protein-protein interaction (PPI) network of the intersecting targets was built using the STRING database. KEGG pathway enrichment analysis was performed using the DAVID v6.8 database. The results of GO and KEGG analyses were visualized using the ggplot2 and paletteer packages in R 4.4.2. Molecular docking of the main components of FZSJF and Osimertinib with MMP9 and TIMP3 was performed and visualized using AutoDock Vina, PyMOL Win, and Discovery Studio 2019.

#### 3.1.2 Analysis of the prognostic value of MMP9 and TIMP3 in lung cancer based on the TCGA database

Lung cancer patient data were obtained from the TCGA database. Data processing and analysis were performed using the Limma, pheatmap, and stringr packages in R 4.4.2. The Wilcoxon test was used to determine the expression levels of MMP9 and TIMP3 in lung cancer patients. Volcano plots of MMP9 and TIMP3 expression in lung cancer patients were generated using the ggplot2 and ggVolcano packages. Perform Spearman correlation analysis between MMP9 and TIMP3, and generate a scatter plot with a fitted line. Paired analysis was performed to assess differences in MMP9 and TIMP3 expression between normal individuals and lung cancer patients. Subsequently, baseline clinical information of lung cancer patients from the TCGA database was retrieved. Patients were divided into MMP9 high- and low-expression groups and TIMP3 high- and low-expression groups. Kaplan-Meier (K-M) curves were plotted, and survival comparisons were performed using the survival and survminer packages in R 4.4.2.

### 3.2 Cell experiments

#### 3.2.1 Cell culture

Complete culture medium was prepared using RPMI Medium 1640 supplemented with 10% fetal bovine serum (FBS) and penicillin-streptomycin (10,000 U/mL) for culturing parental H1975 cells. Osimertinib powder was dissolved in DMSO to prepare a stock solution. H1975OR cells were cultured in RPMI Medium 1640 containing 10% FBS, penicillin-streptomycin (10,000 U/mL), and 1 μmol/L osimertinib. When the cells reached the logarithmic growth phase, they were trypsinized and passaged or used for subsequent experiments. Cells were maintained in a humidified incubator at 37 °C with 5% CO_2_.

#### 3.2.2 Establishment and identification of H1975OR cells

Osimertinib powder was diluted to a concentration of 100 nmol/L and used as the starting concentration for continuous induction of H1975N cells. The concentration was gradually increased to 1 μmol/L over approximately two months. After maintaining the cells at this concentration for two weeks, cells that stably proliferated in the presence of the drug were identified as the resistant cell line and used in subsequent experiments. Colony formation assay, CCK-8 assay, wound healing assay (Wound healing rate (%) = (Scratch area at 0h − Scratch area at time point)/Scratch area at 0h × 100%), and Transwell assay were employed to verify successful induction of drug resistance.

#### 3.2.3 Synergy analysis

H1975N and H1975OR cells were treated with varying concentrations of FZSJF, osimertinib, or the combination of FZSJF and osimertinib. The half-maximal inhibitory concentration (IC_50_) of osimertinib in H1975OR cells was determined using the CCK-8 assay. The combination index (CI) was calculated using CompuSyn software to evaluate the synergistic effect and determine the optimal combination ratio of FZSJF and osimertinib.

#### 3.2.4 Colony formation assay

Cells in the logarithmic growth phase were trypsinized and seeded into 6-well plates at approximately 800 cells per well. After 9–10 days of culture, the culture medium was aspirated, and the wells were washed twice with PBS. Then, 2 mL of 4% paraformaldehyde fixative was added to each well, and the wells were gently agitated. After 20 minutes of fixation, the fixative was aspirated, and the wells were washed 3 times with PBS. Subsequently, 1 mL of crystal violet staining solution was added to each well. After 20 minutes of staining, the staining solution was removed, and the wells were washed 3 times with PBS. Finally, images were captured.

#### 3.2.5 CCK-8 assay

Cells in the logarithmic growth phase were trypsinized and seeded into 96-well plates at approximately 3,000 cells per well. After 24 hours of culture, the culture medium was aspirated, and the cells were treated with the indicated agents. At 24, 48, and 72 hours post-treatment, the medium was aspirated, and the CCK-8 working solution was added to each well. The plates were then incubated in the cell culture incubator for 3 hours in the dark. Finally, the optical density (OD) was measured at 562 nm using a microplate reader.

#### 3.2.6 Transwell assay

Matrigel was thawed at 4 °C for 12 hours. The Matrigel was then diluted with basal medium at a 1:7 ratio. A 50 μL aliquot of the diluted Matrigel was added to the upper chamber of each Transwell insert. The 24-well plate with the inserts was placed in a 37 °C cell culture incubator for 4 hours to allow the Matrigel to solidify. Cells in the logarithmic growth phase were trypsinized, the supernatant was discarded, and the cells were resuspended in serum-free 1640 medium. The cell suspension was seeded into the upper chambers at a density of 3 × 10^4^ cells per well, and 700 μL of complete medium was added to the lower chambers. At 12, 24, and 48 hours, the medium in both the upper and lower chambers was aspirated. The cells were fixed with 4% paraformaldehyde for 20 minutes, stained with crystal violet solution for 20 minutes, and then the staining solution was removed. The wells were washed three times with PBS, and images were captured.

#### 3.2.7 WB assay

BCA protein determination: After treatment, cells were collected and lysed on ice for 15 minutes using ice-cold RIPA lysis buffer containing protease inhibitors. The lysate was centrifuged, and the supernatant was collected. The supernatant was then added to a 96-well plate, followed by the addition of the BCA working solution. The plate was incubated at 37 °C in the dark for 30 minutes. The optical density (OD) was measured at 562 nm using a microplate reader. A concentration curve was calculated using Excel 2021, and protein quantification was performed to ensure equal loading in subsequent steps. Finally, SDS-PAGE sample loading buffer was added, and the samples were boiled for 10 minutes to denature the proteins.

Electrophoresis and transfer: Stacking and separating gels were prepared for protein separation by SDS-PAGE vertical electrophoresis. The PVDF membrane was activated with methanol, and proteins were transferred from the gel to the PVDF membrane using a wet transfer method.

Immunoreaction and development: After transfer, the membrane was blocked with 5% non-fat dry milk for 3 hours at room temperature to block non-specific sites. The membrane was then incubated with primary antibodies diluted in antibody diluent overnight at 4 °C. After washing with TBST, the membrane was incubated with the corresponding HRP-conjugated secondary antibody for 1 hour at room temperature. Following removal of unbound secondary antibody by TBST washing, the membrane was covered with ECL chemiluminescent substrate, and images were captured using a chemiluminescence imaging system with appropriate exposure times.

WB quantification: After chemiluminescence imaging, band densities were quantified using ImageJ software. The optical density of each target protein band was normalized to that of the corresponding GAPDH band, and the relative protein expression was expressed as the ratio of target protein to GAPDH. All experiments were performed independently in triplicate.

#### 3.2.8 qPCR experiment

##### 3.2.8.1 mRNA extraction

The culture medium in 6-well plates was aspirated, and the wells were washed twice with PBS. Then, 350 μL of lysis buffer (Buffer QLS) was added to each well, and the plate was gently shaken to ensure uniform distribution of the buffer over the cell surface. After standing for 7 minutes, 350 μL of anhydrous ethanol was added to each well, and the mixture was thoroughly mixed by pipetting. The resulting solution was transferred to a Quick RNA Mini Column and centrifuged. The filtrate was discarded, 700 μL of Buffer QWB was added to the column, and the column was centrifuged. After discarding the filtrate, the adsorption column of the Quick RNA Mini Column was placed into a new 2.0 mL collection tube and centrifuged. The adsorption column was then transferred to a new RNase-free tube, and 40 μL of DEPC water was added to the center of the column. After standing at room temperature for 3 minutes, the column was centrifuged for 3 minutes to elute the RNA. The eluted RNA was stored at −80 °C.

##### 3.2.8.2 Quantification and reverse transcription

The mRNA samples were removed from the –80 °C freezer and thawed on ice. Quantification was performed using a NanoDrop spectrophotometer. First, 1 μL of nuclease-free water was added to the measurement pedestal, and the pedestal was wiped clean with lens paper. Then, another 1 μL of nuclease-free water was added to the pedestal, and the RNA measurement mode was selected for blanking. Subsequently, 1 μL of each sample was loaded sequentially, and the OD260/OD280 ratio (expected to be between 1.8 and 2.1, indicating good RNA purity) and RNA concentration were recorded. Based on the measured concentrations, the RNA samples were normalized to equal concentrations using nuclease-free water. SuperMix and gDNA remover were mixed at a 4:2 ratio, and the samples were then placed in a thermal cycler for reverse transcription under the following conditions: 42 °C for 15 min, followed by 85 °C for 5 s.

##### 3.2.8.3 Amplification

SYBR^®^ Green and forward/reverse primers were mixed and added to the PCR reaction plate. The reaction conditions were set as follows: Hold Stage: 95 °C for 30 seconds; PCR Stage: 95 °C for 5 seconds, 60 °C for 30 seconds, for 40 cycles; Melt Curve Stage: 60 °C for 30 seconds, 95 °C for 5 seconds. The primer sequences are shown in **Table 1**.

**Table 1:** Primer sequences.

Gene	Primer sequence (5′-3′)
GAPDH	Forward Primer: CTAGGCGCTCACTGTTCTCT
	Reverse Primer: GCCCAATACGACCAAATCCGT
MMP9	Forward primer: ATGTACCGCTTCACTGAGGG
	Reverse primer: TCAGGGCGAGGACCATAGAG
TIMP3	Forward primer: ACCGAGGCTTCACCAAGATG
	Reverse primer: CCATCATAGACGCGACCTGT.

##### 3.2.8.4 qPCR quantification

The relative mRNA expression levels of MMP9 and TIMP3 were calculated using the 2^-^ΔΔCt method, with GAPDH as the internal reference gene. Each sample was run in triplicate, and the average Ct values were used for calculation. The fold change was normalized to the control group.

#### 3.2.9 IF assay

Cells were trypsinized and seeded onto culture plates containing glass coverslips. After the cells had adhered and reached an appropriate density, the culture medium was removed. The cells were fixed with pre-chilled 4% paraformaldehyde, permeabilized with Triton X-100, and then blocked with 5% BSA for 1 hour at room temperature to block non-specific sites. The cells were incubated with specific primary antibodies overnight at 4 °C. After PBS washing, the cells were incubated with the corresponding fluorochrome-conjugated secondary antibodies for 1 hour at room temperature in the dark. Nuclei were counterstained with DAPI. Finally, the coverslips were mounted in an anti-fluorescence quenching medium, and images were acquired using a laser-scanning confocal microscope.

For quantitative analysis of immunofluorescence staining, at least three random fields per coverslip were captured using a laser-scanning confocal microscope with identical acquisition parameters. The mean fluorescence intensity of each image was measured using ImageJ software. The relative fluorescence intensity was normalized to the control group after background subtraction. Each experiment was independently repeated at least three times.

#### 3.2.10 Lentiviral transduction to establish the CAF cell model with stable TIMP3 silencing

1. Determination of multiplicity of infection (MOI) and cell plating:

Determine the required MOI of the virus according to the kit instructions and preliminary experiments. Cells in the logarithmic growth phase were trypsinized and prepared into a single-cell suspension. 4 × 10^4^ cells per well were seeded into a 24-well plate. According to the kit instructions, three wells were set for the control group, three wells for the empty vector group, and three wells for the experimental group. The 24-well plate was then placed in a 37 °C, 5 % CO_2_ incubator and cultured overnight to allow cell attachment.

2. Lentiviral transduction:

Take out the virus stock, the NC group virus stock, and the transfection solution, and place them on ice to thaw slowly. Remove the 24-well plate and aspirate the culture medium from the wells of the NC and experimental groups. Add the prepared virus suspension to each corresponding well (500 μL per well). Then quickly close the lid, label properly, and return the plate to the incubator.

3. Medium change and observation:

24 hours after transduction, remove the 24-well plate, aspirate the culture medium from each well, and add 500 μL of antibiotic-free 1640 complete medium to each well. Continue culturing the plate in the incubator for another 48 hours.

4. Puromycin selection:

Prepare a culture medium containing 10% FBS, 90% RPMI Medium 1640, and 2 μg·mL^-^¹ puromycin. Aspirate the medium from the 24-well plate, wash the plate twice with PBS, and add the new puromycin-containing medium. Change the medium every 24 hours. Observe the transduction efficiency under an inverted fluorescence microscope. Transduction is considered successful when the cells in the blank control group are completely dead.

#### 3.2.11 Cell co-culture

##### 3.2.11.1 Seeding in the lower chamber

H1975N cells in the logarithmic growth phase were trypsinized, and the cell density was adjusted to 2 × 10^5^ cells/mL. 2 mL of the H1975N cell suspension was added to each well of the lower chamber of the cell culture insert plate, ensuring uniform distribution of the cells. The plate was placed in an incubator and cultured overnight to allow the cells to adhere fully.

##### 3.2.11.2 Seeding in the upper chamber

The next day, after confirming that the H1975N cells were well attached, the upper chamber of the cell culture insert was prepared. The density of CAFs was adjusted to 2 × 10^5^ cells/mL. 1 mL of the CAFs cell suspension was added to the upper chamber of the experimental group. In comparison, 1 mL of complete culture medium was slowly added to the upper chamber of the control group. The plate was gently agitated to ensure uniform distribution of the cells.

##### 3.2.11.3 Co-culture

The plate was returned to a 37 °C, 5 % CO_2_ incubator and co-cultured for 48 hours. After co-culture, protein or mRNA was extracted from the cells in the lower chamber, and changes in the expression of target proteins and mRNAs were detected by WB or qPCR.

### 3.3 Animal experiments

#### 3.3.1 Preparation of rat medicated serum

All rats were acclimated for one week before the experiment. Equivalent doses for rats were calculated based on body surface area conversion. The intragastric dose of FZSJF was approximately 2.275g·kg^-1^, and the dose of osimertinib for rats was approximately 2.5mg·kg^-1^ according to the manufacturer’s instructions. The rats received intragastric administration twice daily (1 mL each time) for 10 consecutive days. Two hours after the final intragastric administration, the rats were anesthetized with tribromoethanol, and blood was collected from the abdominal aorta. The blood was centrifuged, and the upper serum layer was collected. The serum was then placed in a 56 °C water bath for 30 minutes. After filtration through a 0.22 μm filter, the serum was used to prepare the following complete culture media: control group complete medium (10% control rat medicated serum + 90% 1640 medium + 1% penicillin-streptomycin), osimertinib group complete medium (10% osimertinib rat medicated serum + 90% 1640 medium + 1% penicillin-streptomycin), FZSJF group complete medium (10% FZSJF rat medicated serum + 90% 1640 medium + 1% penicillin-streptomycin), and combination group complete medium (10% combination rat medicated serum + 90% 1640 medium + 1% penicillin-streptomycin). The prepared complete media were stored at 4 °C, and the remaining serum was aliquoted and stored at −80 °C.

#### 3.3.2 UPLC-Q TOF MS/MS

##### 3.3.2.1 Instruments

Ultra-high performance liquid chromatography (UHPLC, U3000+), high-resolution mass spectrometer (QExactive Focus), and a chromatography column (T3, 1.8 μm, 100 × 2.1 mm).

##### 3.3.2.2 Sample preparation of FZSJF

The FZSJF preparation was mixed thoroughly, and 0.5 mL of the preparation was taken. Then, 0.5 mL of ice-cold acetonitrile was added, followed by vortexing for 1 minute. The mixture was centrifuged at 12,000 rpm at room temperature for 5 minutes, and 0.8 mL of the supernatant was transferred to a liquid chromatography vial.

##### 3.3.2.3 Blood sample preparation

The blood sample was mixed well, and 200 μL was transferred into a 1.5 mL Eppendorf tube. Then, 600 μL of methanol: acetonitrile (1:1) was added, and the mixture was vortexed for 60 seconds. After standing at 4 °C for 10 minutes, the sample was centrifuged at 12,000 rpm for 10 minutes. A 700 μL volume of the supernatant was transferred to a new 1.5 mL Eppendorf tube and dried under nitrogen gas. The residue was reconstituted in 100 μL of 60% aqueous methanol, and 80 μL of the supernatant was transferred into an insert vial for subsequent analysis.

##### 3.3.2.4 Mobile phase conditions

Column temperature: 40 °C; injection volume: 2 μL; mobile phase A: 0.1% formic acid in water; mobile phase B: 0.1% formic acid in acetonitrile.

##### 3.3.2.5 Comprehensive spectral identification

To further determine the compound composition of the herbal formula and eliminate redundant structures, the obtained QE mass spectrometry data were uploaded to Progenesis QI for searching against an in-house formula mass spectrometry database. The in-house database was compiled using MySQL and contained information such as chemical structures, names, and mass spectral fragments, stored in SDF format. Compounds were identified based on mass spectral fragment scores, and those with a score >30 were selected.

#### 3.3.3 Nude mouse xenograft model establishment and gavage

##### 3.3.3.1 First batch of nude mice

All nude mice were first acclimatized for one week. During this period, H1975N, H1975OR, and CAFs cells were expanded in large quantities. Cells in the logarithmic growth phase were harvested by trypsinization, centrifuged, and resuspended in PBS. The nude mice were then randomly divided into three groups using a random number table: H1975N group (n=6), H1975OR group (n=6), and H1975N+CAFs group (n=6). Mice in the H1975N group were inoculated subcutaneously in the axilla with 3×10^6^ H1975N cells. Mice in the H1975OR group received 3×10^6^ H1975OR cells. Mice in the H1975N+CAFs group were co-inoculated with 3×10^6^ H1975N cells and 1×10^6^ CAFs cells (15). Tumor volume and body weight were measured every two days for all groups. Tumor volume was calculated using the formula: V = L × W² × 0.5 (V = tumor volume, L = tumor long diameter, W = tumor short diameter). Tumors formed approximately 8 weeks after inoculation. Mice were then anesthetized with tribromoethanol (0.2 mL·10g^-^¹) and sacrificed by cervical dislocation. Tumor tissues were dissected and photographed. Part of each tumor was snap-frozen in liquid nitrogen for subsequent protein extraction, and the remaining part was fixed in 4% paraformaldehyde for paraffin embedding and histopathological analysis.

##### 3.3.3.2 Second batch of nude mice

Using a random number table, nude mice were divided into four groups (n=6 per group): control group, low-dose Fuzheng Sanjie Formula (FZSJF) group, medium-dose FZSJF group, and high-dose FZSJF group. Each mouse was inoculated subcutaneously in the axilla with 3×10^6^ H1975OR cells. Approximately 8 weeks later, when tumors had formed, the equivalent doses of FZSJF for nude mice were calculated based on body surface area. The administration doses for each group were as follows: 1) control group: daily intragastric administration of pure water; 2) low-dose group: 1.138 g·kg^-^¹·day^-^¹; 3) medium-dose group: 2.275 g·kg^-^¹·day^-^¹; 4) high-dose group: 4.550 g·kg^-^¹·day^-^¹. All groups received intragastric administration twice daily for 14 consecutive days. Two hours after the last administration, mice were anesthetized with tribromoethanol (0.2 mL·10g^-^¹) and sacrificed by cervical dislocation (16). The remaining procedures were the same as described in section 2.3.3 (1).

##### 3.3.3.3 Third batch of nude mice

After the completion of the second batch experiment, another batch of nude mice was randomly divided into four groups (n=6 per group) using a random number table: control group, osimertinib group, FZSJF group, and combination group. Each mouse was inoculated subcutaneously in the axilla with 3×10^6^ H1975OR cells. Approximately 8 weeks later, when tumors had formed, the equivalent doses for nude mice were calculated based on body surface area. The administration doses for each group were as follows: 1) control group: daily intragastric administration of pure water; 2) osimertinib group: approximately 5 mg·kg^-^¹ according to the osimertinib package insert; 3) FZSJF group: 4.550 g·kg^-^¹ of FZSJF; 4) combination group: osimertinib 5 mg·kg^-^¹ plus FZSJF 4.550 g·kg^-^¹. The remaining procedures were the same as described in section 2.3.3 (1).

#### 3.3.4 WB assay of animal tissues

Tissue pieces were washed 2–3 times with pre-chilled PBS and then cut into small fragments. The fragments were placed into grinding tubes, and lysis buffer was added at a ratio of 200 μL per 20 mg of tissue. Three to six zirconia beads were added to each grinding tube, and the tissues were ground using a tissue grinder until fully lysed. The homogenate was then centrifuged at 12,000 rpm for 10 minutes at 4 °C, and the supernatant was collected. The remaining steps were the same as those described in section 2.2.6.

### 3.4 Statistical methods

For comparisons between two groups, if the data were normally distributed and had homogeneous variances, an independent-samples t-test was used; otherwise, the Mann-Whitney U test was used. For comparisons among three or more groups, if the data followed a normal distribution with homogeneous variances, one-way analysis of variance (ANOVA) was used, followed by Bonferroni correction for post-hoc pairwise comparisons. If the data followed a normal distribution but had unequal variances, one-way ANOVA was used, followed by Dunnett’s T3 test for pairwise comparisons. If the data did not follow a normal distribution, the Kruskal-Wallis rank-sum test was used for intergroup comparisons, and the Mann-Whitney U test for pairwise comparisons. For comparisons of measurements at multiple time points across multiple groups, repeated measures ANOVA was applied. All statistical analyses were performed using SPSS 27.0.

### 3.5 Quality control and statistical considerations in experimental design

#### 3.5.1 Randomization and blinding

All animal experiments were performed under a double-masked design, in which the personnel responsible for drug administration and those responsible for tumor measurement, body weight recording, and subsequent histological analyses were mutually blinded to group allocation. Sample codes were encrypted during the detection phase and were not unblinded until after data analysis was completed.

#### 3.5.2 Sample size determination

The sample size for animal experiments was calculated based on the mean and standard deviation of tumor volumes obtained from preliminary pilot experiments and relevant literature (15), using G*Power software. The calculation indicated that at least 5 mice per group were required; considering a 10% attrition rate, the final number was set at 6 mice per group. For cell experiments, at least three replicate wells were set per experiment to ensure statistical power. All cell experiments were performed in three independent biological replicates, with at least three technical replicate wells per experiment, to ensure statistical power.

#### 3.5.3 Inclusion and exclusion criteria (for animal experiments)

Inclusion criteria were: SPF-grade male BALB/c nude mice, aged 4 weeks, weighing 16–20 g, in good health after a 1-week acclimatization period. Exclusion criteria were (1): failure to form measurable tumors (volume <50 mm³) within 4 weeks post-inoculation (2); body weight loss >20% during the experiment, or development of severe infection or moribund status (3); tumor ulceration or necrosis that interfered with accurate measurement. Animals meeting any exclusion criterion were removed from the study and replaced.

## 4 Results

### 4.1 UPLC-Q-TOF-MS/MS, network pharmacology, and data mining results

#### 4.1.1 UPLC-Q-TOF-MS/MS

**Figure 1:**
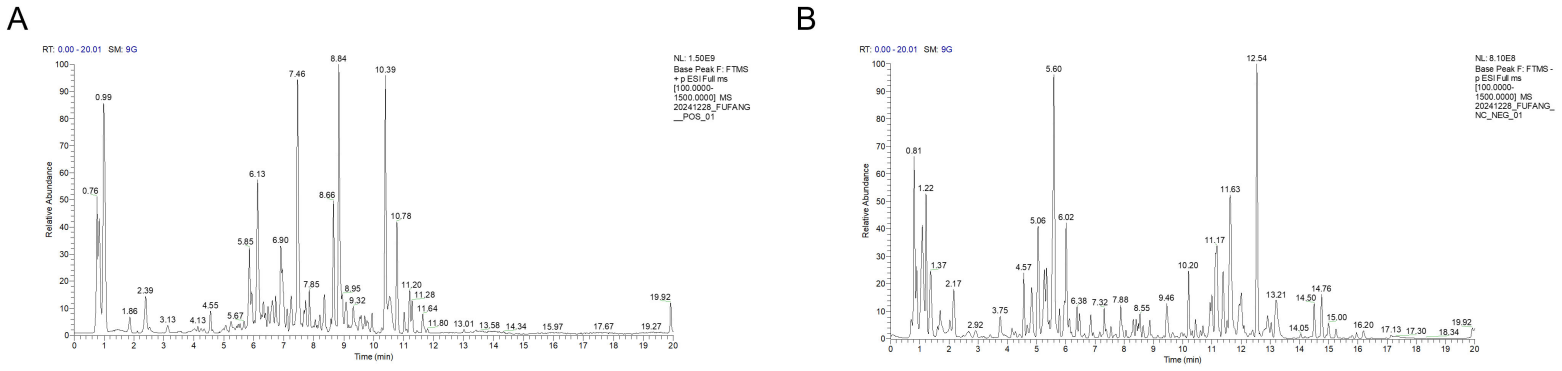
Representative base peak chromatograms acquired under positive ion mode **(A)** and negative ion mode **(B)** by comprehensive spectral component identification analysis.

To elucidate the main chemical composition of FZSJF, the chemical components and characteristics of the formula were comprehensively and systematically analyzed using HPLC-Q-TOF MS technology. The base peak chromatograms obtained under positive and negative electrospray ionization modes are shown in **Figure 1**. Finally, 29 major chemical constituents were identified from FZSJF, as listed in **Table 2**.

**Table 2:** Main chemical constituents of FZSJF identified by UPLC-Q-TOF-MS/MS.

No.	Name	Formula	Adducts	Fragmentation Score	Mass Error	m/z	RT (min)
1	Platycodin D	C57H92O28	M+FA-H	94	-0.92	1223.5691	7.05
2	Afzelin	C21H20O10	M+H	92	-1.34	433.1123	6.16
3	Astragalin	C21H20O11	M-H	91.2	-0.21	447.0932	5.9
4	Scutellarin	C21H18O12	M-H	93.6	-0.23	461.0724	6.13
5	Baicalin	C21H18O11	M+H	87.8	-1.32	447.0916	6.58
6	Vitexin	C21H20O10	M+FA-H	86.8	-0.41	477.1037	8.02
7	Curdione	C15H24O2	M+H	88.5	-1.73	219.1739	10.32
8	Emodin-1-O-β-D-glucopyranoside	C21H20O10	M-H	87.8	-0.69	431.0981	6.16
9	lobetyol	C14H18O3	M+FA-H	85.5	-0.77	279.1236	7.57
10	Curcumenol	C15H22O2	M+FA-H	85.4	-1.03	279.1599	11.03
11	naringenin	C15H12O5	M-H2O-H	82.2	-0.86	253.0504	9.18
12	Protocatechuic Acid	C7H6O4	M-H	80.8	-7.65	153.0182	5.71
13	Eriodictyol	C15H12O6	M+FA-H	79.1	-0.8	287.0559	7.73
14	p-coumaric acid	C9H8O3	M-H	79.4	-6.95	163.0389	6.24
15	Scutellarein	C15H10O6	M+H	79.9	-2.34	287.0543	7.23
16	Skullcapflavone II	C19H18O8	M+H	77.5	-1.88	375.1067	9.37
17	chrysin	C15H10O4	M-H	73.7	-1.04	253.0504	11.16
18	Bergapten	C12H8O4	M+H	70.3	-1.24	217.0493	9.32
19	Emodinanthrone	C15H12O4	M-H2O-H	72.9	-3.41	237.0548	8.98
20	apigenin	C15H10O5	M-H	70.4	-1.5	269.0451	6.86
21	kaempferol	C15H10O6	M-H	70.9	-3.39	285.0395	6.6
22	luteolin	C15H10O6	M-H	67.5	-0.44	285.0403	8.63
23	Scutebarbatine A	C32H34N2O7	M+FA-H	66.2	0.62	603.2352	10.46
24	Crysophanol	C15H10O4	M-H	67.5	-1.06	253.0504	9.04
25	quercetin	C15H10O7	M-H	63.9	-1.41	301.035	7.79
26	Syringic Acid	C9H10O5	M-H2O-H	63.7	-5.69	179.0339	5.51
27	Wogonin	C16H12O5	M+FA-H	62.1	-0.22	329.0666	7.77
28	Laminarin	C18H32O16	M+FA-H	65.8	1.6	549.1671	0.9
29	Rhein	C15H8O6	M-H	62.8	-0.01	283.0248	8.88

#### 4.1.2 Network pharmacology and molecular docking of FZSJF

**Figure 2:**
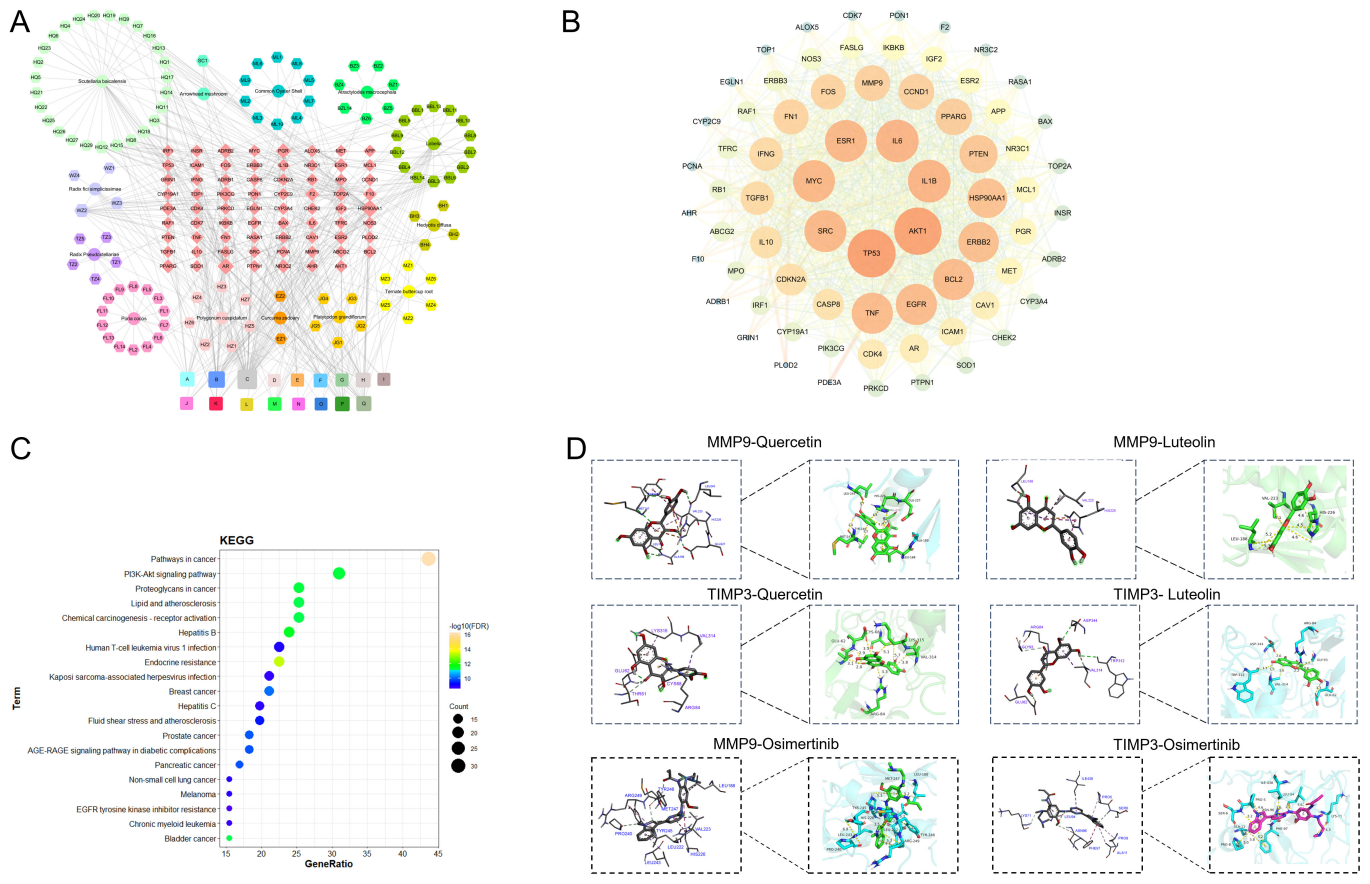
Network pharmacology and molecular docking diagrams of FZSJF. **(A)** Herb-component-disease-target network of FZSJF; **(B)** PPI analysis of overlapping targets among FZSJF, NSCLC, CAFs, and osimertinib resistance; **(C)** KEGG pathway enrichment analysis of overlapping targets among FZSJF, NSCLC, CAFs, and osimertinib resistance; **(D)** Schematic diagrams of molecular docking between main active components of FZSJF and Osimertinib with MMP9 and TIMP3.

Integrating the results of UPLC-Q-TOF-MS/MS with the TCMSP and BATMAN-TCM databases, network pharmacology analysis of FZSJF was performed. The results showed that MMP9 is one of the key targets through which FZSJF acts on NSCLC (**Figures 2A–B**). KEGG pathway analysis revealed that FZSJF may influence NSCLC treatment by modulating pathways such as Pathways in cancer, Non-small cell lung cancer, and EGFR tyrosine kinase inhibitor resistance (**Figure 2C**). Molecular docking demonstrated that the main active components of FZSJF and Osimertinib exhibited very high docking scores with MMP9 and TIMP3 (**Figure 2D**, **Table 3**).

**Table 3:** Molecular docking scores of the main active components of FZSJF and Osimertinib with MMP9 and TIMP3.

Compound	MMP9 docking score	TIMP3 docking score
Quercetin	-9.83	-8.73
Luteolin	-10.09	-8.81
Osimertinib	-9.04	-8.2

#### 4.1.3 Analysis of MMP9 and TIMP3 expression in lung cancer based on the TCGA database

**Figure 3:**
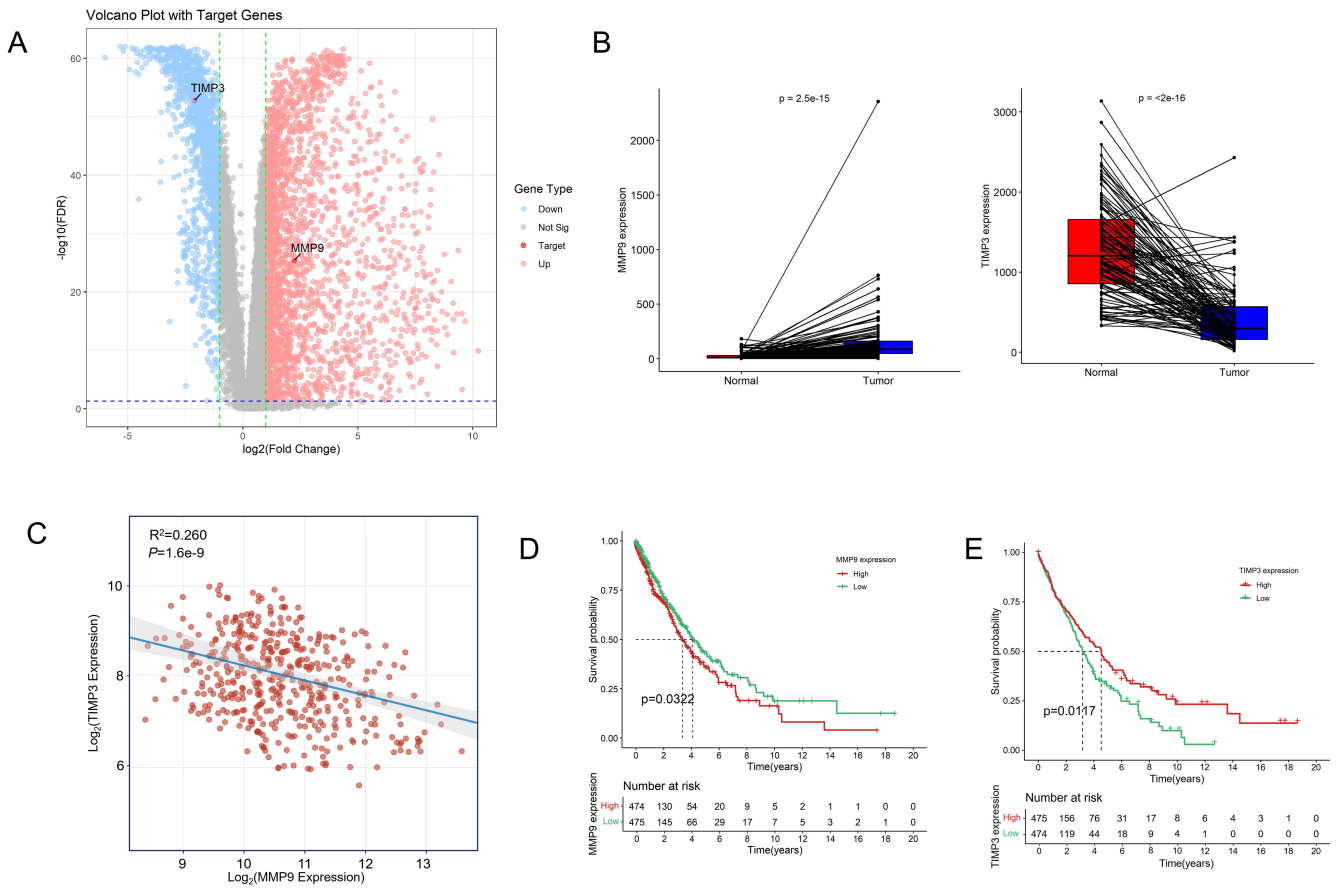
Analysis of MMP9 and TIMP3 expression in lung cancer patients based on the TCGA database. **(A)** Volcano plot showing TIMP3 and MMP9 expression in tumor tissues; **(B)** Paired analysis of MMP9 and TIMP3 expression in normal versus tumor tissues; **(C)** Spearman correlation analysis between MMP9 and TIMP3. **(D)** Kaplan-Meier curve analysis of the effect of MMP9 expression level on OS in lung cancer patients; **(E)** Kaplan-Meier curve analysis of the effect of TIMP3 expression level on OS in lung cancer patients.

Expression data of MMP9 and TIMP3 in lung cancer patients were extracted from the TCGA database. Differentially expressed genes between the normal and tumor groups were screened using the criteria of FDR < 0.05 and |log_2_FoldChange| > 0.585. The results showed that MMP9 and TIMP3 were differentially expressed between the normal and tumor groups (**Figures 3A, B**). Moreover, a significant correlation was observed between the expression levels of MMP9 and TIMP3 (R² = 0.260, P = 0.6 × 10^-9^) (**Figure 3C**). Furthermore, lung cancer patients with high MMP9 expression had significantly shorter OS than those with low expression (**Figure 3D**). In contrast, patients with high TIMP3 expression had significantly longer OS than those with low expression (**Figure 3E**).

### 4.2 Cellular experiment results

#### 4.2.1 Establishment of osimertinib-resistant H1975 cell model (H1975OR)

**Figure 4:**
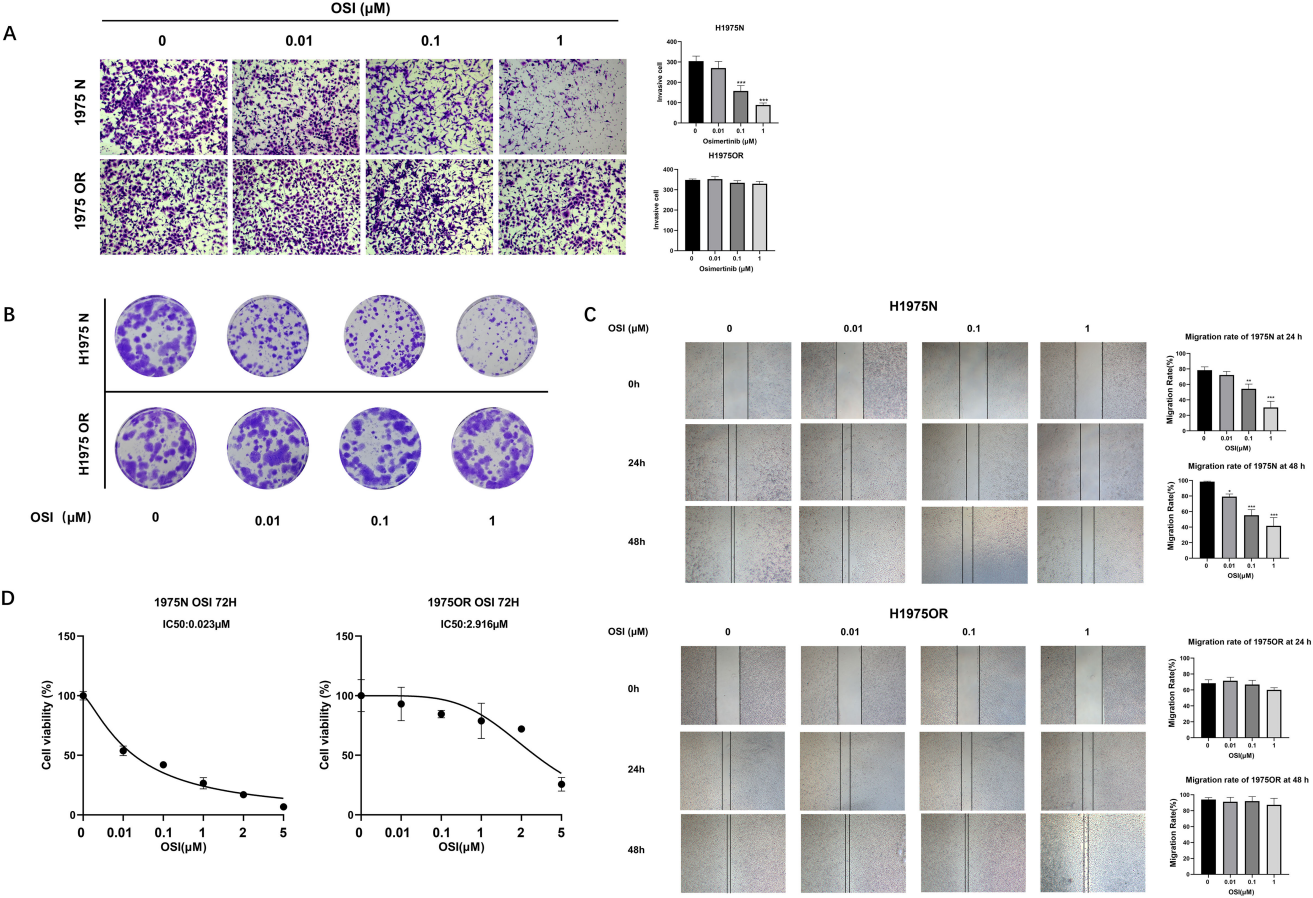
Validation of H1975OR cell model establishment **(A)** Representative images of Transwell invasion assay (n=3) showing the invasive capacity of H1975N and H1975OR cells after 72 hours of exposure to different concentrations of osimertinib; the right panel shows the corresponding statistical bar chart. **(B)** Colony formation assay detecting the inhibitory effect of different concentrations of osimertinib on colony formation of H1975N and H1975OR cells at 72 hours. **(C)** Wound healing assay (n=3) assessing the effect of different concentrations of osimertinib on the migration of H1975N and H1975OR cells; the right panel shows the corresponding statistical bar chart. **(D)** CCK8 assay (n=3) measuring the cell viability of H1975N and H1975OR cells after 72 hours of exposure to different concentrations of osimertinib. Error bars: SD. *P < 0.05, **P < 0.01, ***P < 0.001.

In this study, H1975N cells were stimulated with gradually increasing concentrations of osimertinib. After approximately two months, resistant cells were successfully induced, and the final osimertinib concentration was maintained at 1 μmol·L^-1^. The invasive capacity and colony formation ability of osimertinib-sensitive and osimertinib-resistant cells were assessed by Transwell invasion assay (**Figure 4A**), wound healing assay (n=3) (**Figure 4B**), and colony formation assay (n=3) (**Figure 4C**). The results showed that osimertinib at different concentrations inhibited the invasive ability of parental H1975 cells but had no significant inhibitory effect on H1975OR cells. The CCK-8 assay (n=3) showed that the IC_50_ of osimertinib was 0.023 μmol·L^-1^ for H1975N cells and 2.916 μmol·L^-1^ for H1975OR cells (**Figure 4D**). These results collectively indicate that an osimertinib-resistant NSCLC cell line was successfully established.

2.2.3 Synergy analysis

#### 4.2.2 Synergy analysis result

**Figure 5:**
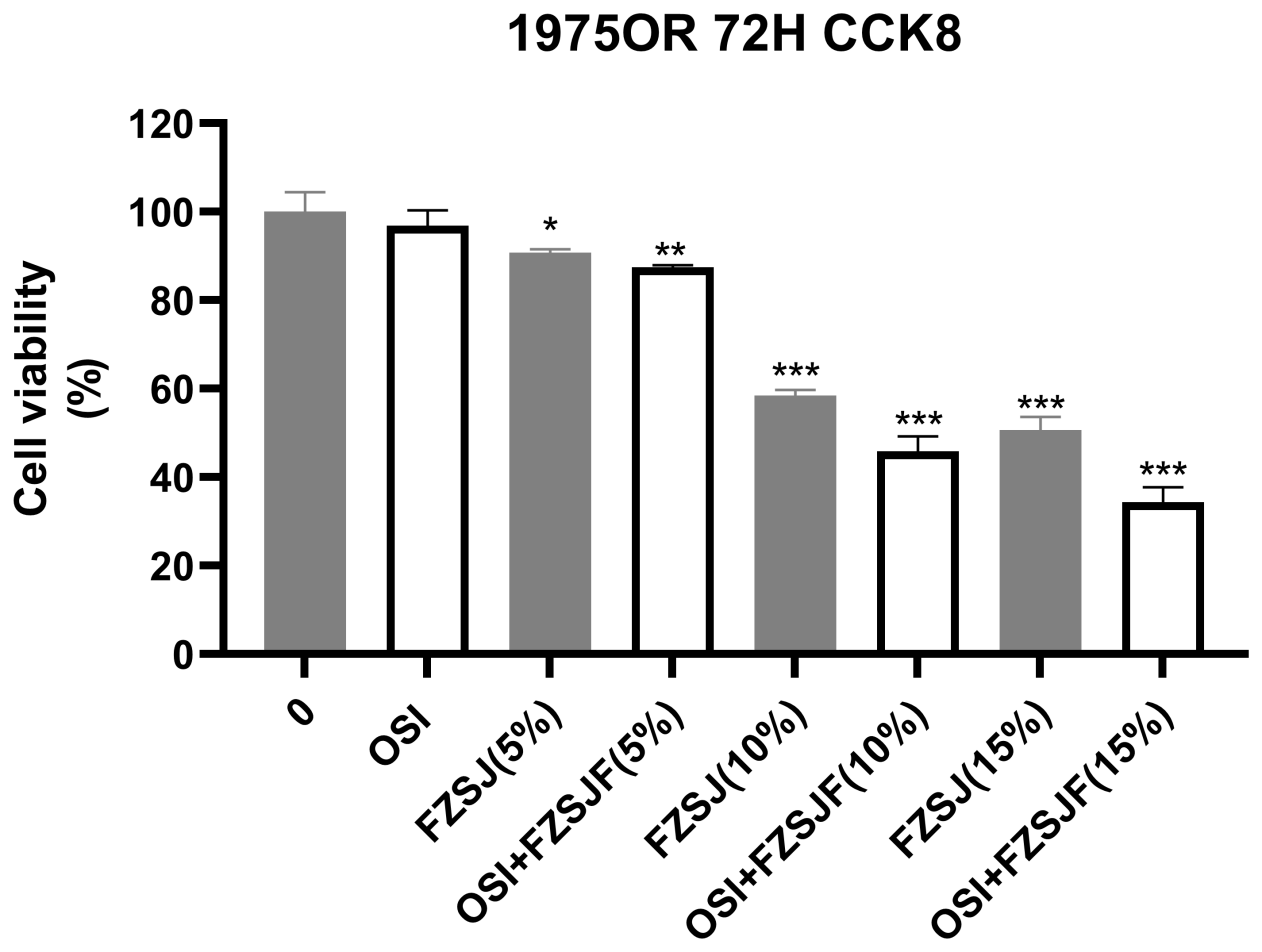
The survival rates of H1975OR cells after 72-hour treatment with osimertinib, FZSJF, or their combination were determined by CCK-8 assay. *P<0.05, **P<0.01, ***P<0.001.

H1975OR cells were treated with blank, FZSJF-containing, or osimertinib-containing serum, and IC_50_ values were determined via CCK-8 assay. The combination of osimertinib and FZSJF markedly inhibited H1975OR cell growth (**Figure 5**). CompuSyn-derived CI values confirmed synergy between the two agents (CI<1), with the combination of osimertinib and 10% FZSJF-containing serum showing the optimal synergistic effect (**Table 4**).

**Table 4:** CI for the combination of osimertinib and FZSJF.

OSI	FZSJF(%)	CI
+	5%	0.65
+	10%	0.53
+	15%	0.66

#### 4.2.3 Demonstration that FZSJF exerts anti-osimertinib-resistant NSCLC effects

**Figure 6:**
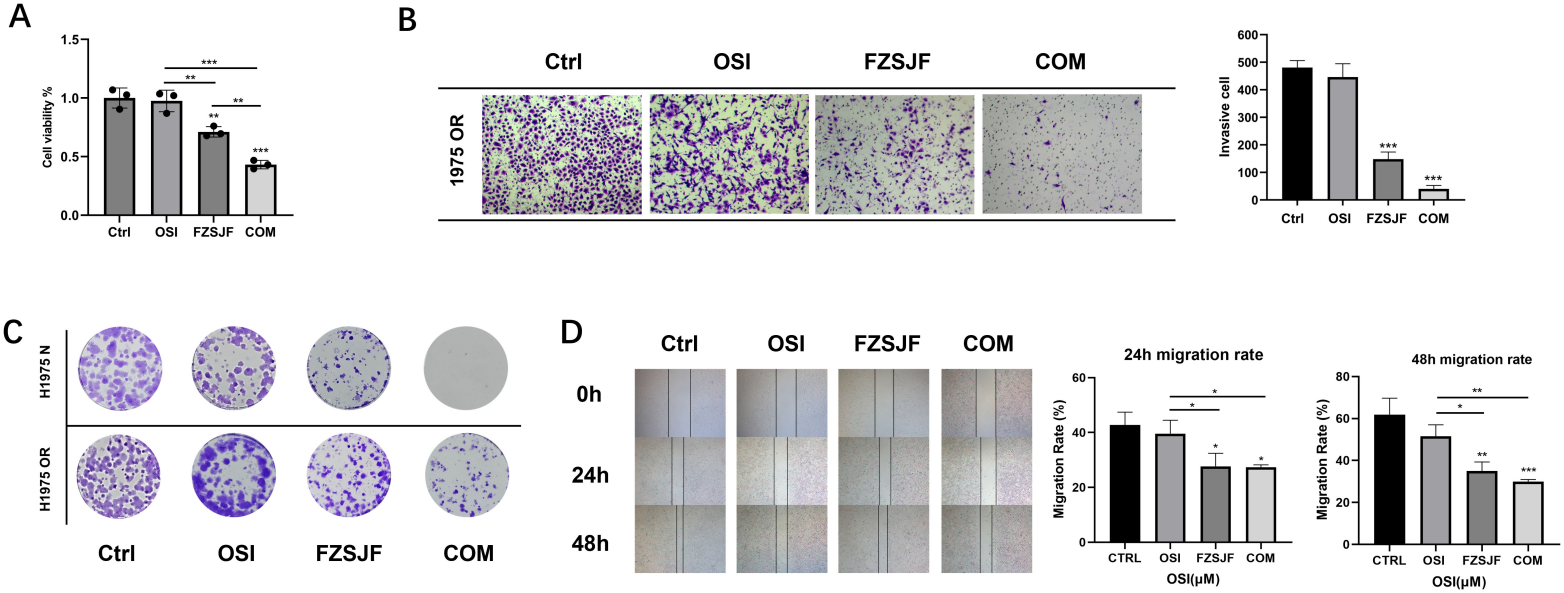
Phenotypic assays of the effects of FZSJF alone and in combination with osimertinib on H1975OR cells. **(A)** Cell viability of H1975OR cells after 72 hours of treatment assessed by CCK-8 assay (n=3); **(B)** Invasion of H1975OR cells after 72 hours of treatment evaluated by Transwell invasion assay (n=3); **(C)** Colony formation of H1975OR cells after 8 days of treatment determined by colony formation assay; **(D)** Invasion of H1975OR cells assessed by wound healing assay (n=3) at 0, 24, and 48 hours after treatment. Error bars: SD. *P < 0.05, **P < 0.01, ***P < 0.001.

H1975OR cells were treated with complete media from the control group, osimertinib group, FZSJF group, and combination group. The CCK-8 assay showed that both the FZSJF group and the combination group significantly inhibited H1975OR cell growth (**Figure 6A**). The Transwell invasion assay (**Figure 6B**), colony formation assay (**Figure 6C**), and wound healing assay (**Figure 6D**) all demonstrated that FZSJF alone or in combination with osimertinib inhibited the invasive ability of H1975OR cells.

#### 4.2.4 Differential expression of MMP9 and TIMP3 in H1975N and H1975OR cells

**Figure 7:**
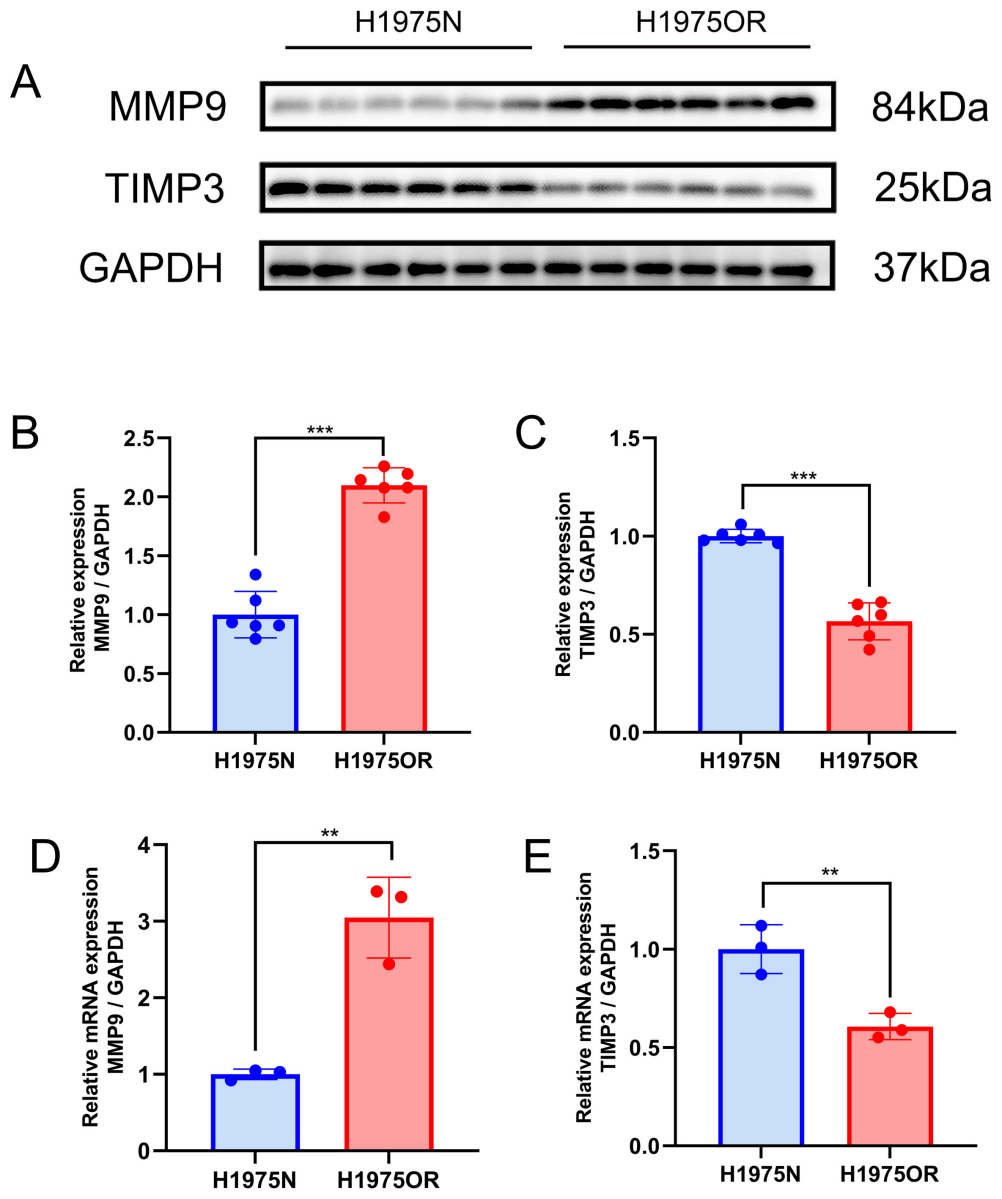
Expression of MMP9 and TIMP3 at protein (n=6) and mRNA levels (n=3) in H1975N and H1975OR cells. **(A)** Representative WB bands; **(B)** Bar chart showing MMP9 protein expression detected by WB; **(C)** Bar chart showing TIMP3 protein expression detected by WB; **(D)** Bar chart showing MMP9 mRNA expression detected by qPCR; **(E)** Bar chart showing TIMP3 mRNA expression detected by qPCR. Note: Error bars: SD; *P < 0.05, **P < 0.01, ***P < 0.001; For qPCR analysis, n = 3 represents three independent biological replicates.

WB and qPCR analyses revealed that H1975OR cells exhibited high MMP9 expression at both the protein and mRNA levels (**Figures 7A, B, D**), whereas TIMP3 expression was low at both the protein and mRNA levels (**Figures 7A, C, E**).

#### 4.2.5 Identification and characterization of CAFs

**Figure 8:**
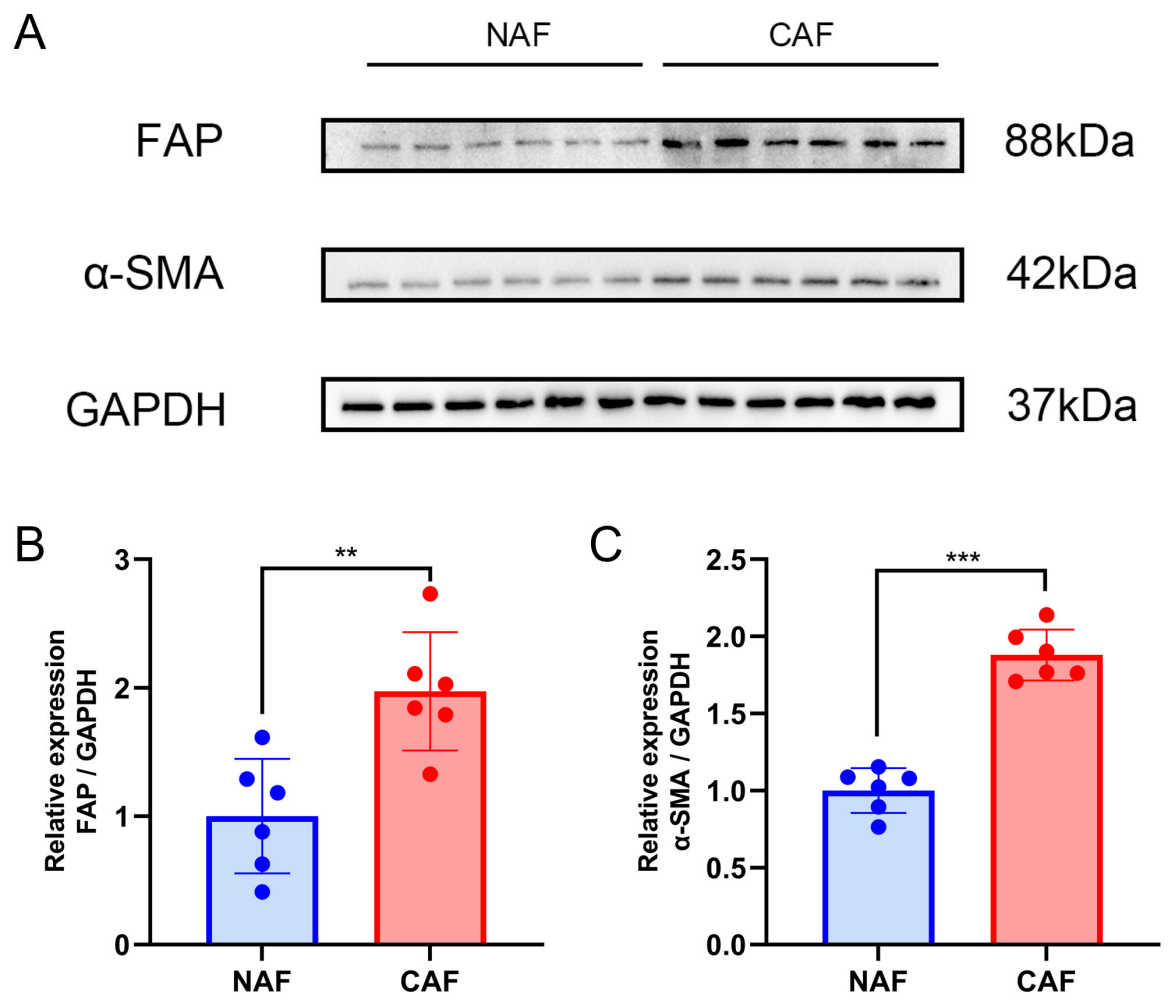
Protein expression levels of FAP and α-SMA in CAFs and NAFs (n=6). **(A)** Representative WB bands; **(B)** Bar chart showing FAP protein expression detected by WB; **(C)** Bar chart showing α-SMA protein expression detected by WB.

To confirm the identity of CAFS induced from HFL1 cells by TGF-β stimulation, the expression of established CAF markers was examined. CAFs were characterized by WB for fibroblast activation protein (FAP) and alpha-smooth muscle actin (α-SMA). Western blot results showed that the expression of FAP and α-SMA was upregulated in CAFs compared with NAFs (**Figures 8A–C**), which partially confirmed the successful induction of CAFs.

#### 4.2.6 Differential expression of MMP9 and TIMP3 in NAF and CAF cells

**Figure 9:**
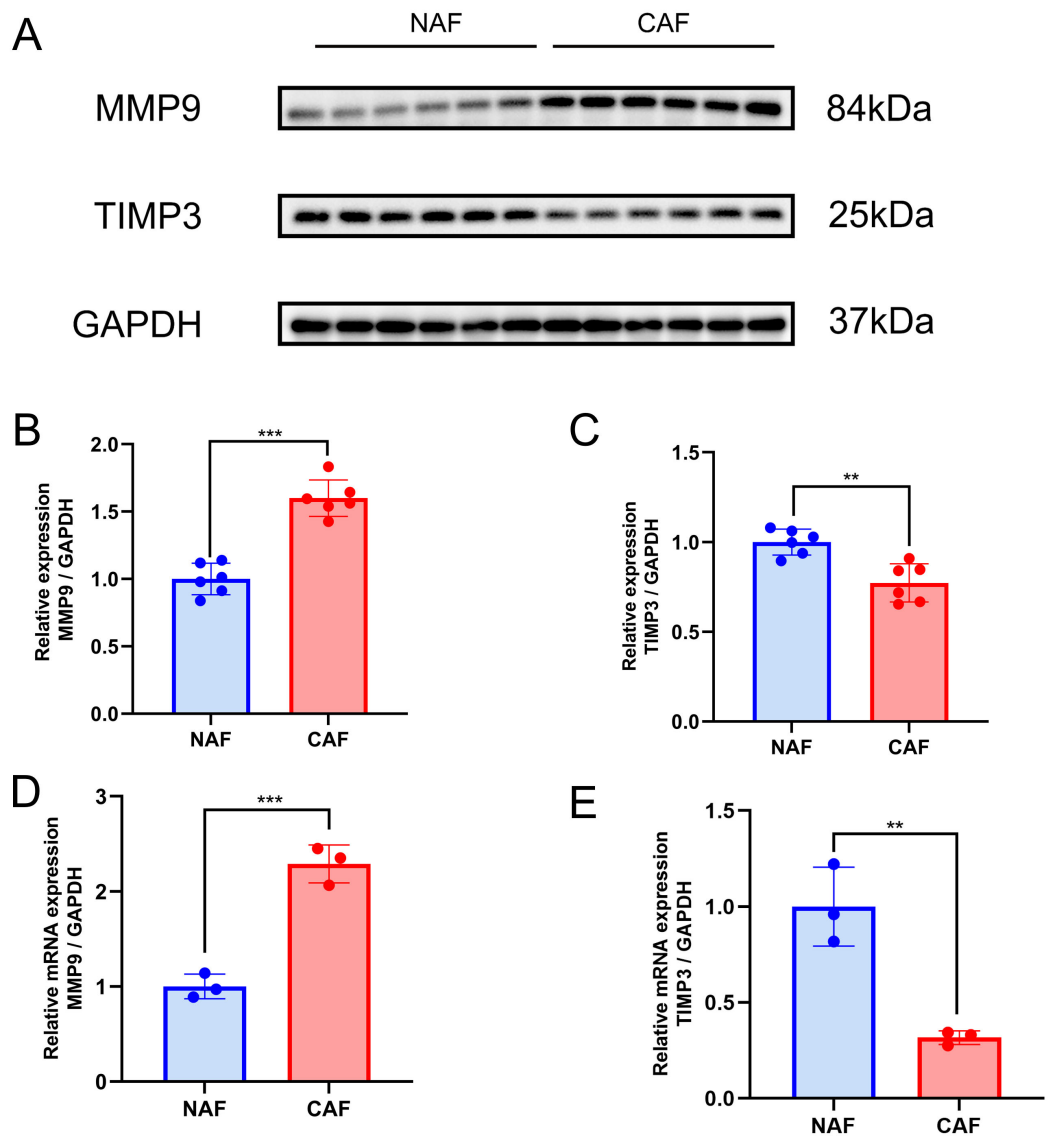
Expression of MMP9 and TIMP3 at protein (n=6) and mRNA levels (n=3) in CAFs and NAFs. **(A)** Representative WB bands; **(B)** Bar chart showing MMP9 protein expression detected by WB; **(C)** Bar chart showing TIMP3 protein expression detected by WB; **(D)** Bar chart showing MMP9 mRNA expression detected by qPCR; **(E)** Bar chart showing TIMP3 mRNA expression detected by qPCR. Error bars: SD. *P < 0.05, **P < 0.01, ***P < 0.001; For qPCR analysis, n = 3 represents three independent biological replicates.

WB and qPCR analyses revealed that CAFS exhibited high MMP9 expression at both the protein and mRNA levels (**Figures 9A, B, D**), whereas TIMP3 expression was low at both the protein and mRNA levels (**Figures 9A, C, E**).

#### 4.2.7 Construction of CAFs with stable TIMP3 silencing

**Figure 10:**
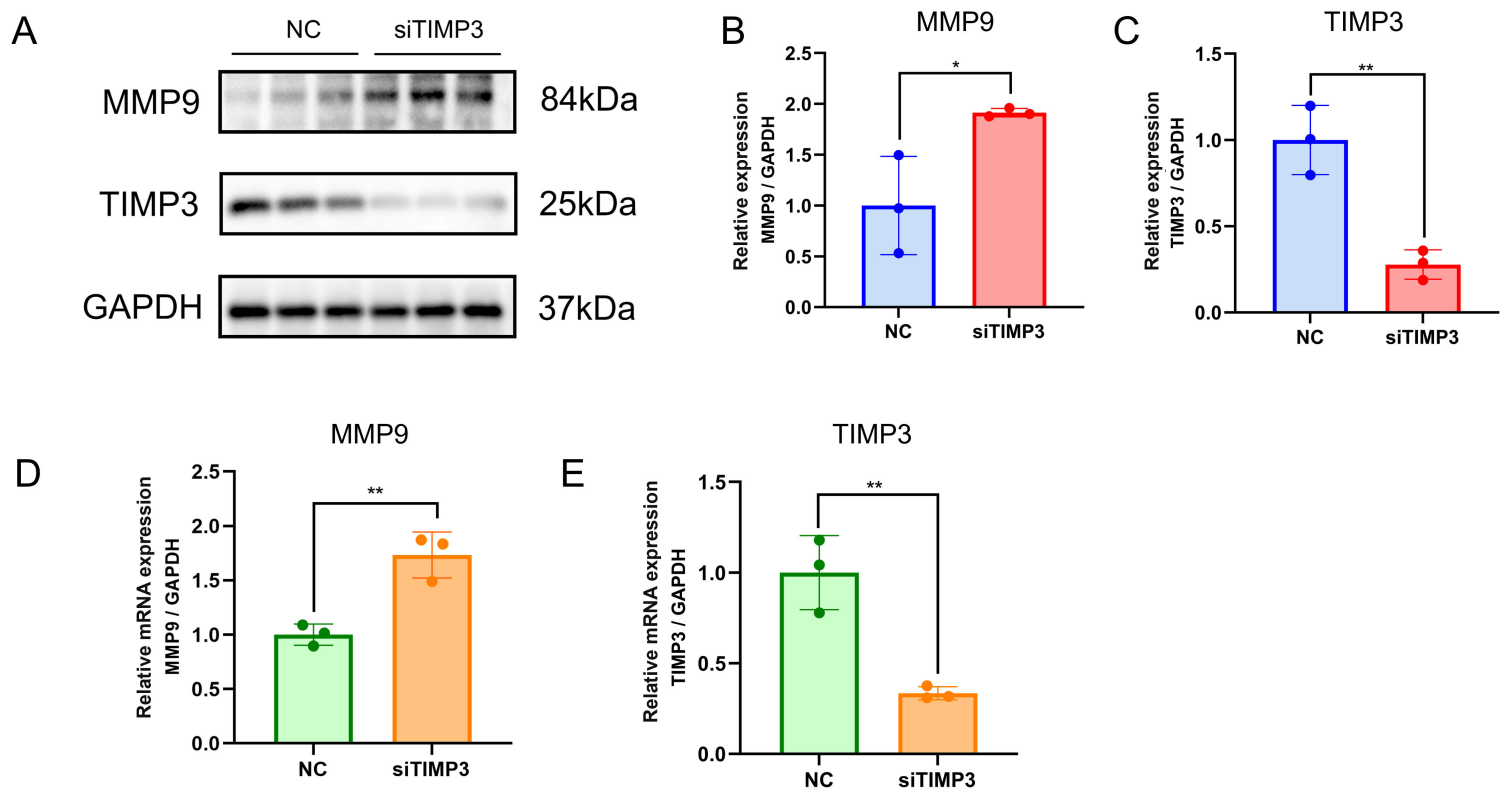
MMP9 and TIMP3 protein (n=3) and mRNA expression levels (n=3) in CAFs with stable TIMP3 silencing. **(A)** Western blot analysis of MMP9 and TIMP3 protein expression in CAFs with stable TIMP3 silencing; **(B)** Bar graph of MMP9 protein expression; **(C)** Bar graph of TIMP3 protein expression; **(D)** Bar graph of MMP9 mRNA expression; **(E)** Bar graph of TIMP3 mRNA expression. Error bars: SD. *P < 0.05, **P < 0.01, ***P < 0.001; For qPCR analysis, n = 3 represents three independent biological replicates.

To further elucidate the mechanism by which the CAFs-TIMP3-MMP9 axis influences EMT, this study established CAFs with stable TIMP3 silencing using lentiviral transduction. We examined the mRNA and protein expression levels of TIMP3 and MMP9 after TIMP3 silencing. The results showed that CAFs with stable TIMP3 silencing were successfully constructed. Compared with the control group (NC group), the CAFs in the TIMP3-silencing group (siTIMP3 group) exhibited higher expression levels of MMP9, as shown in **Figures 10A–E**.

#### 4.2.8 Comparison of H1975N cells co-cultured with the NC group and the siTIMP3 group

**Figure 11:**
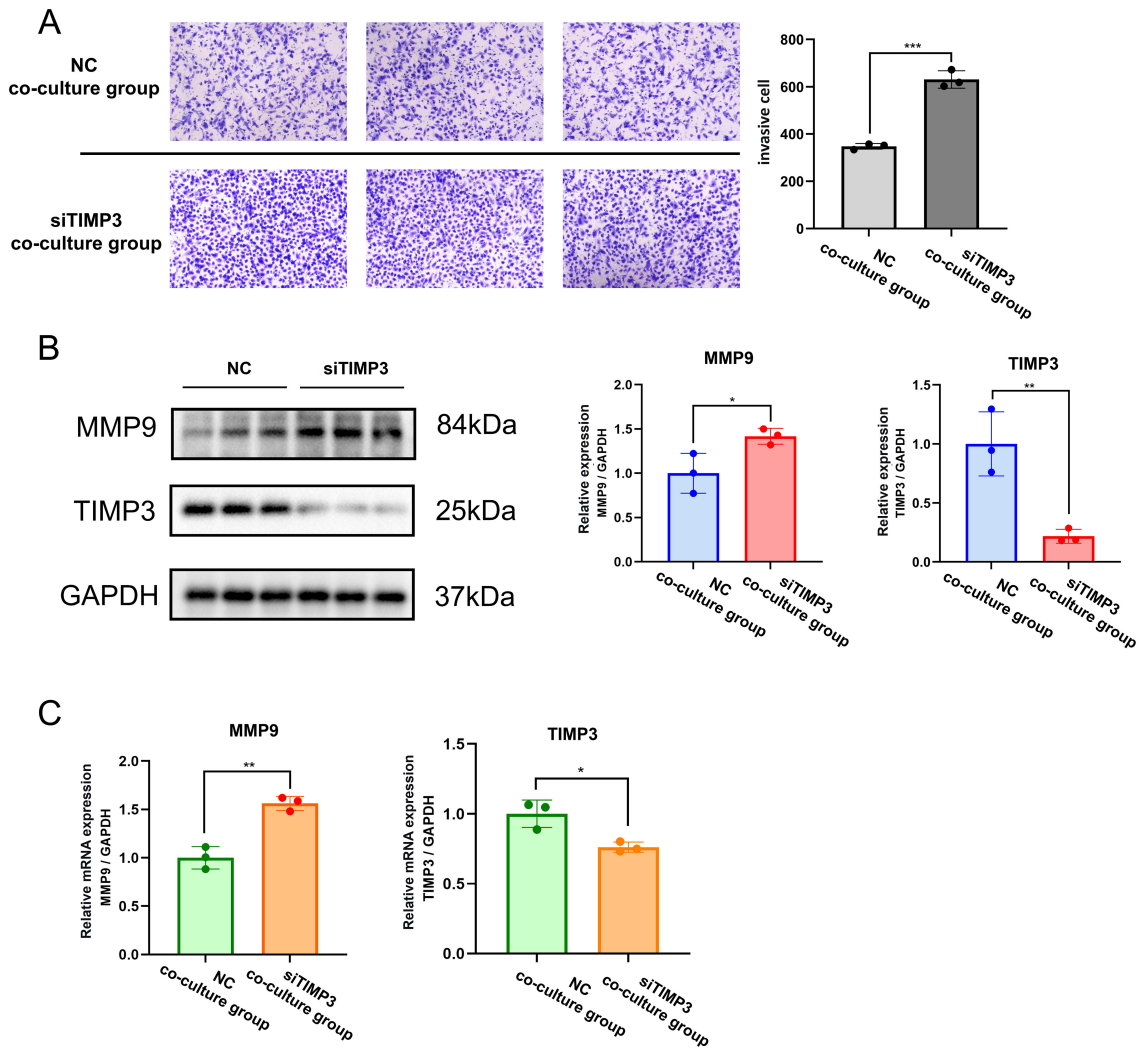
Comparison of H1975N cells co-cultured with CAFs from the NC group and the siTIMP3 group. **(A)** Transwell invasion assay (n=3) of the NC group and siTIMP3 group with corresponding statistical bar graphs; **(B)** WB results (n=3) of H1975N cells co-cultured with CAFs from the NC group and siTIMP3 group; **(C)** qPCR results (n=3) of H1975N cells co-cultured with CAFs from the NC group and siTIMP3 group. Error bars: SD. *P < 0.05, **P < 0.01, ***P < 0.001; For qPCR analysis, n = 3 represents three independent biological replicates.

CAFs from the NC group and the siTIMP3 group were co-cultured with H1975N cells. The invasive ability of H1975N cells after co-culture with the NC group or the siTIMP3 group was assessed using a Transwell invasion assay. The results showed that compared with H1975N cells co-cultured with the NC group, the invasive ability of H1975N cells co-cultured with the siTIMP3 group was enhanced (**Figure 11A**), accompanied by high expression of MMP9 at both protein and mRNA levels (**Figures 11B, C**) and low expression of TIMP3 at both protein and mRNA levels (**Figures 11B, C**).

#### 4.2.9 Regulatory effect of FZSJF on MMP9 and TIMP3 expression in H1975OR cells

**Figure 12:**
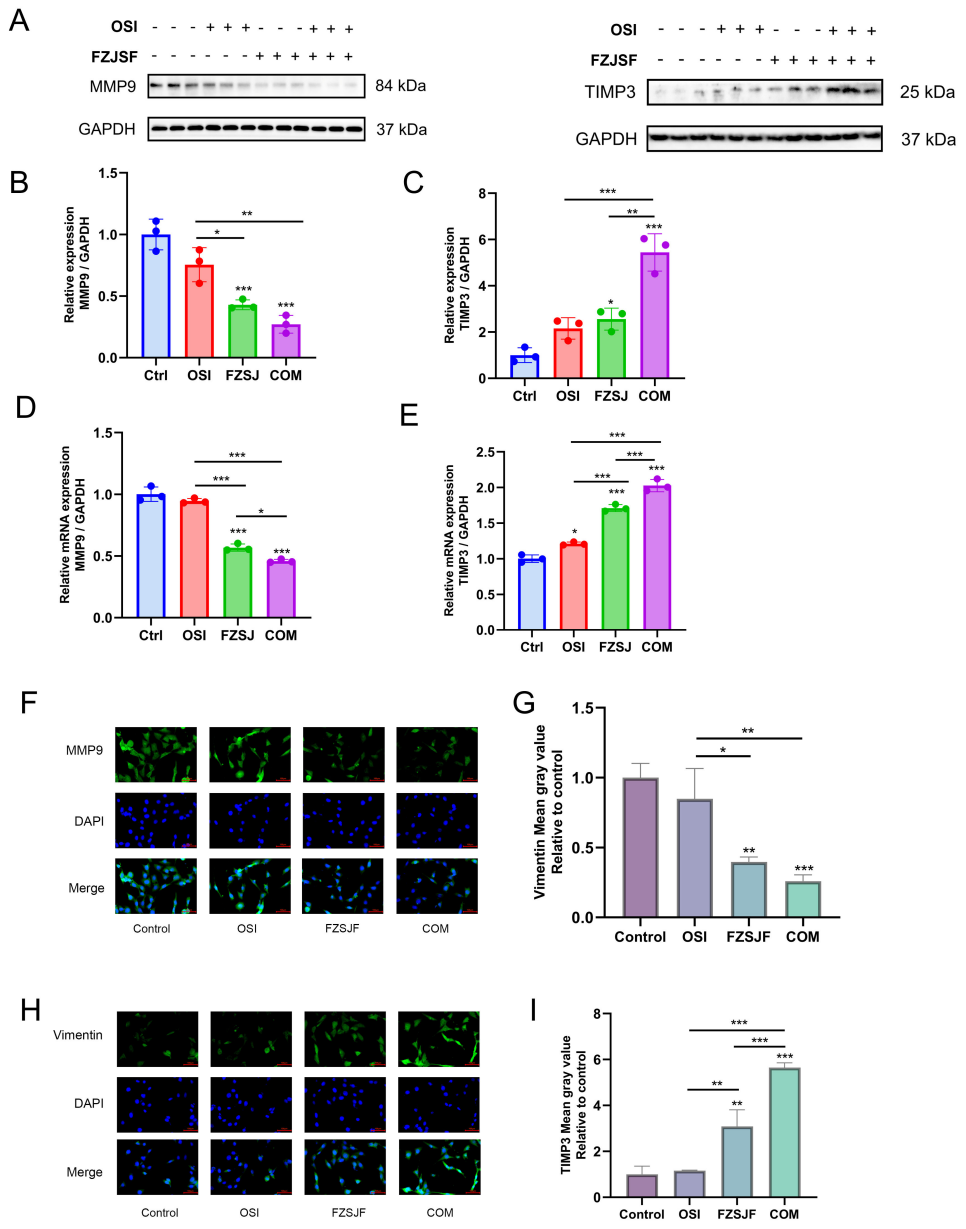
Expression of MMP9 and TIMP3 at protein and mRNA levels in H1975OR cells treated with rat medicated serum detected by WB (n=3), qPCR (n=3), and IF (n=3). **(A)** Representative WB bands; **(B)** Bar chart showing MMP9 protein expression detected by WB; **(C)** Bar chart showing TIMP3 protein expression detected by WB; **(D)** Bar chart showing MMP9 mRNA expression detected by qPCR; **(E)** Bar chart showing TIMP3 mRNA expression detected by qPCR. **(F)** IF images of MMP9; G: Bar chart showing MMP9 IF expression; **(H)** IF images of TIMP3; **(I)** Bar chart showing TIMP3 IF expression. Error bars: SD. *P < 0.05, **P < 0.01, ***P < 0.001; For qPCR analysis, n = 3 represents three independent biological replicates.

H1975OR cells were treated for 48 hours with rat medicated serum from the blank group, osimertinib group, FZSJF group, and combination group. WB, IF, and qPCR detected MMP9 and TIMP3 expression at both the protein and mRNA levels. The results showed that FZSJF alone or in combination with osimertinib upregulated TIMP3 protein and mRNA expression while downregulating MMP9 protein and mRNA expression in H1975OR cells (**Figures 12A–I**).

#### 4.2.10 Regulatory effect of FZSJF on MMP9 and TIMP3 expression in CAFs

**Figure 13:**
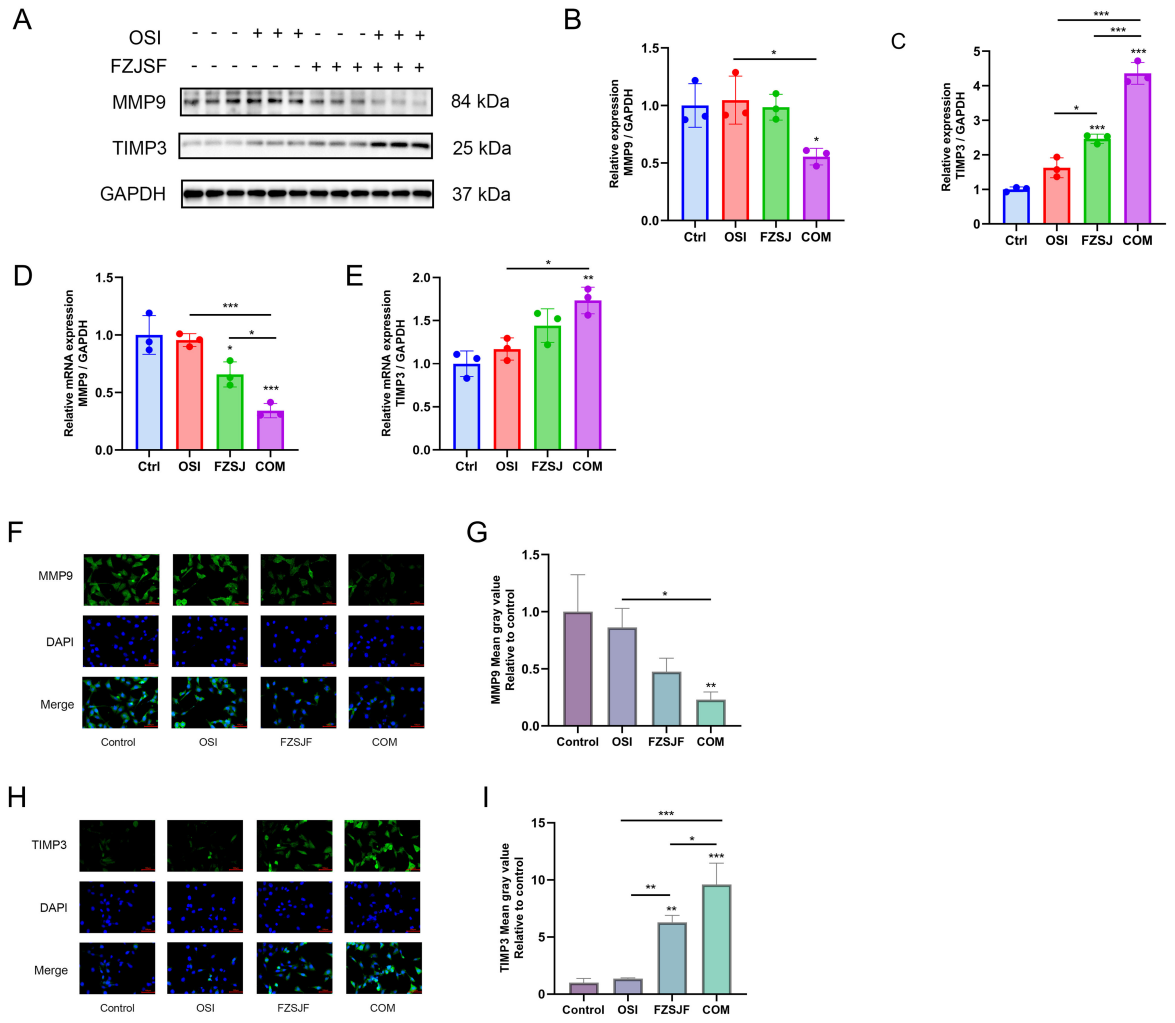
Expression of MMP9 and TIMP3 at protein and mRNA levels in CAFs cells treated with rat medicated serum detected by WB (n=3), qPCR (n=3), and IF (n=3). **(A)** Representative WB bands; **(B)** Bar chart showing MMP9 protein expression detected by WB; **(C)** Bar chart showing TIMP3 protein expression detected by WB; **(D)** Bar chart showing MMP9 mRNA expression detected by qPCR; **(E)** Bar chart showing TIMP3 mRNA expression detected by qPCR. **(F)** IF images of MMP9; **(G)** Bar chart showing MMP9 IF expression; **(H)** IF images of TIMP3; **(I)** Bar chart showing TIMP3 IF expression. Error bars: SD. *P < 0.05, **P < 0.01, ***P < 0.001; For qPCR analysis, n = 3 represents three independent biological replicates.

CAFs were treated for 48 hours with rat medicated serum from the blank group, osimertinib group, FZSJF group, and combination group. WB, IF, and qPCR detected MMP9 and TIMP3 expression at both the protein and mRNA levels. The results showed that FZSJF alone or in combination with osimertinib upregulated TIMP3 protein and mRNA expression while downregulating MMP9 protein and mRNA expression in CAFs (**Figures 13A–I**).

### 4.3 Animal experiment results

#### 4.3.1 H1975N, H1975OR, and H1975N+CAFs groups of nude mice

##### 4.3.1.1 Tumor growth and body weight of nude mice

**Figure 14:**
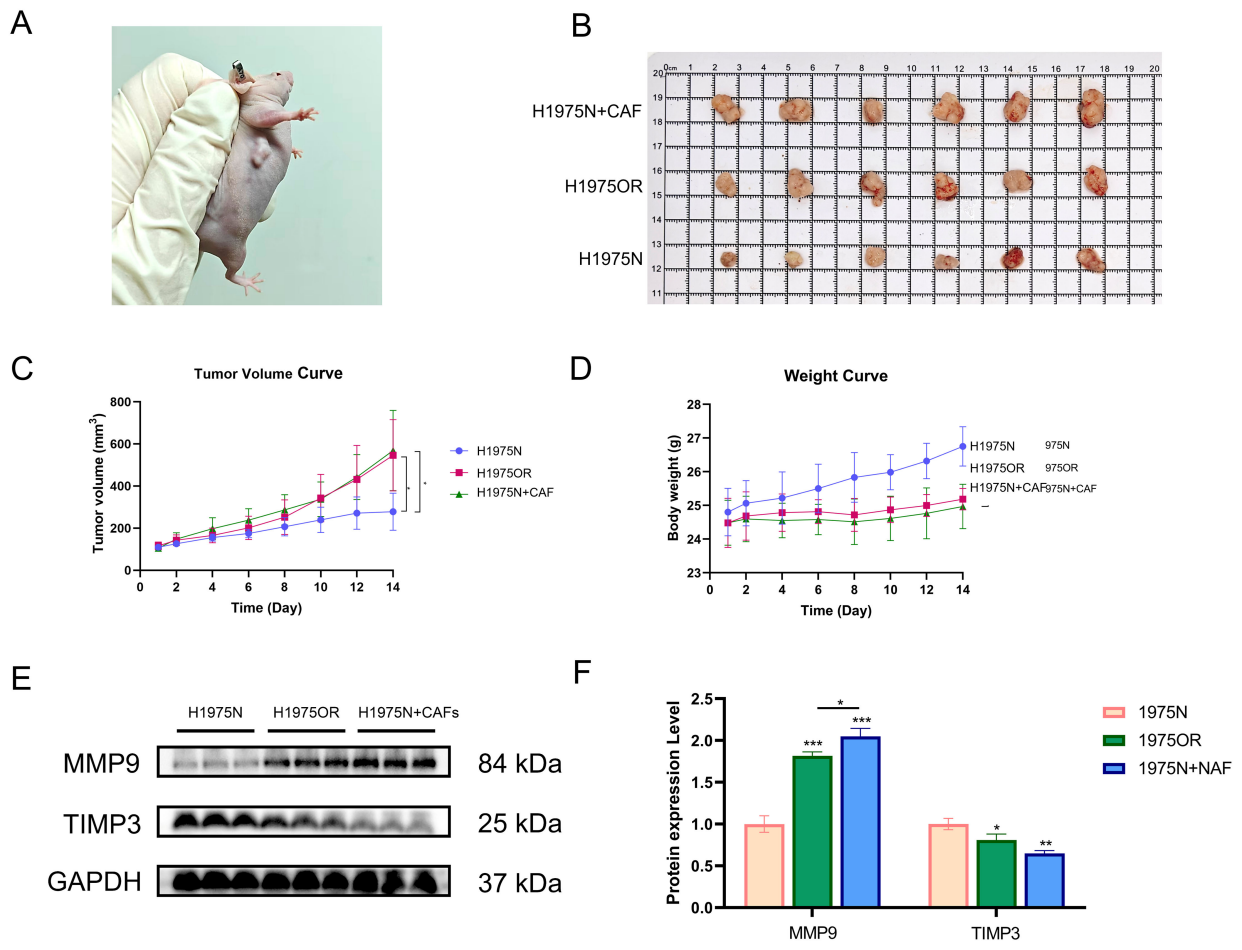
TH1975N, H1975OR, and H1975N+CAFs groups of nude mice. **(A)** Schematic diagram of successful xenograft tumor model establishment; **(B)** Excised tumors on day 14 (n=6); **(C)** Tumor volume growth curves (n=6); **(D)** Body weight change curves (n=6); **(E)** Representative WB bands (n=3); **(F)** Bar charts showing MMP9 and TIMP3 protein expression detected by WB (n=3). Error bars: SD. *P < 0.05, **P < 0.01, ***P < 0.001.

Tumors formed in each group of nude mice at 8 weeks after cell inoculation, as shown in **Figure 14A**. The results indicated that compared with the H1975N group, both the H1975OR and H1975N+CAFs groups exhibited significant differences in tumor volume and body weight, as shown in **Figures 14B–D**.

##### 4.3.1.2 Protein expression levels in the H1975N, H1975OR, and H1975N+CAFs groups

WB revealed high expression of MMP9 and low expression of TIMP3 in the tumor tissues of the H1975OR and H1975N+CAFs groups, as shown in **Figures 14E, F**.

#### 4.3.2 Intervention of the Fuzheng Sanjie Formula in a nude mouse xenograft model of osimertinib-resistant NSCLC

##### 4.3.2.1 Tumor growth and body weight of nude mice

**Figure 15:**
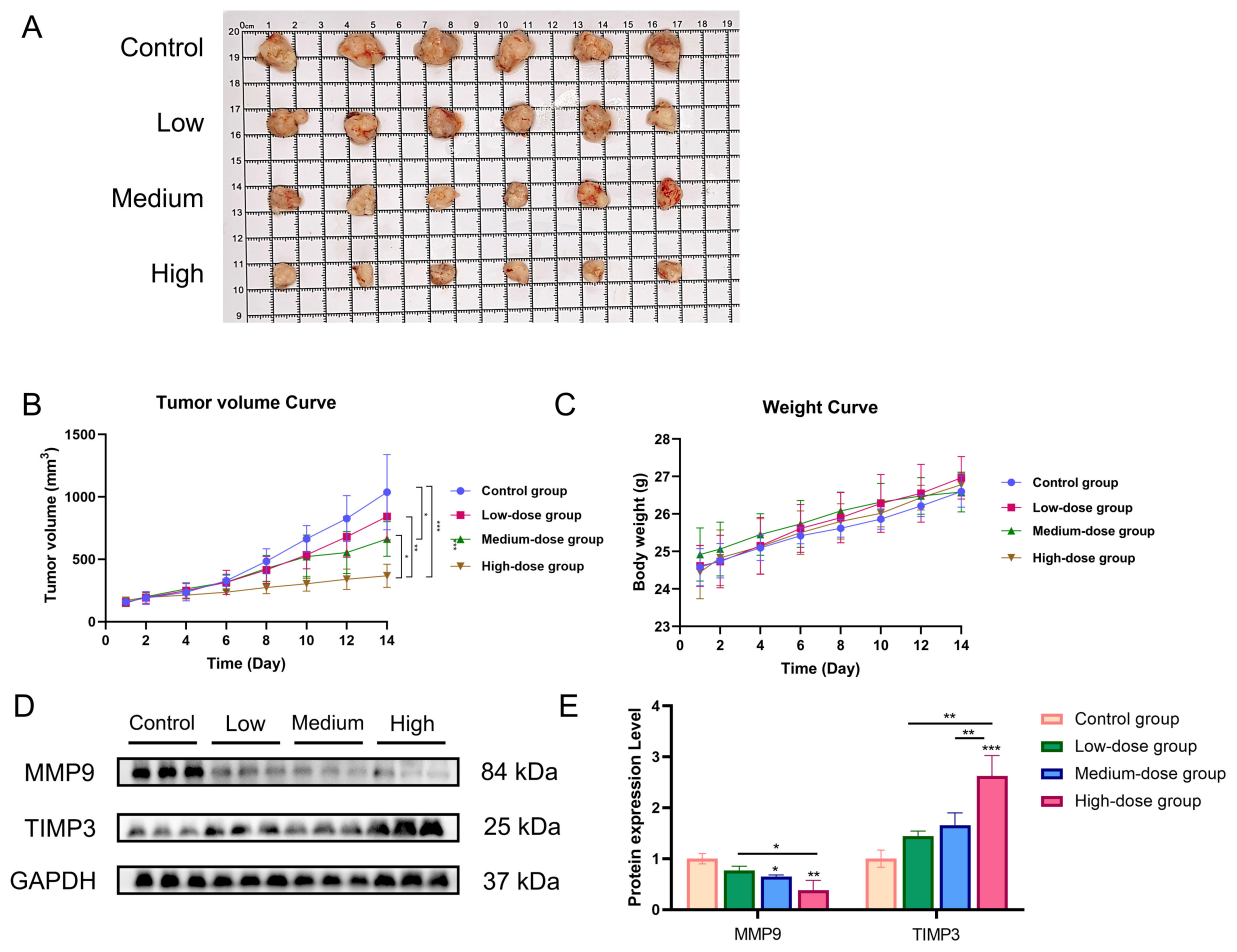
Intervention of the FZSJF in a nude mouse xenograft model of osimertinib-resistant NSCLC. **(A)** Excised tumors on day 14 (n=6); **(B)** Tumor volume growth curves (n=6); **(C)** Body weight change curves (n=6); **(D)** Representative WB bands (n=3); **(E)** Bar charts showing MMP9 and TIMP3 protein expression detected by WB (n=3). Error bars: SD. *P < 0.05, **P < 0.01, ***P < 0.001.

Tumors successfully formed in each group of nude mice approximately 8 weeks after cell inoculation. The results showed that compared with the control group, the medium- and high-dose FZSJF groups exhibited statistically significant differences in tumor volume, with the high-dose group demonstrating the best therapeutic effect, as shown in **Figures 15A, B**. No statistically significant differences in body weight were observed among the groups, as shown in **Figure 15C**. These results indicate that the FZSJF can significantly delay the progression of osimertinib-resistant NSCLC.

##### 4.3.2.2 Protein expression levels of MMP9 and TIMP3 in nude mice

Western blot analysis revealed high expression of TIMP3 in the tumor tissues of the high-dose FZSJF group, and low expression of MMP9 in the tumor tissues of the medium- and high-dose FZSJF groups, as shown in **Figures 15D, E**.

#### 4.3.3 Intervention of the FZSJF combined with osimertinib in a nude mouse xenograft model of osimertinib-resistant NSCLC

##### 4.3.3.1 Tumor growth and body weight in nude mice

**Figure 16:**
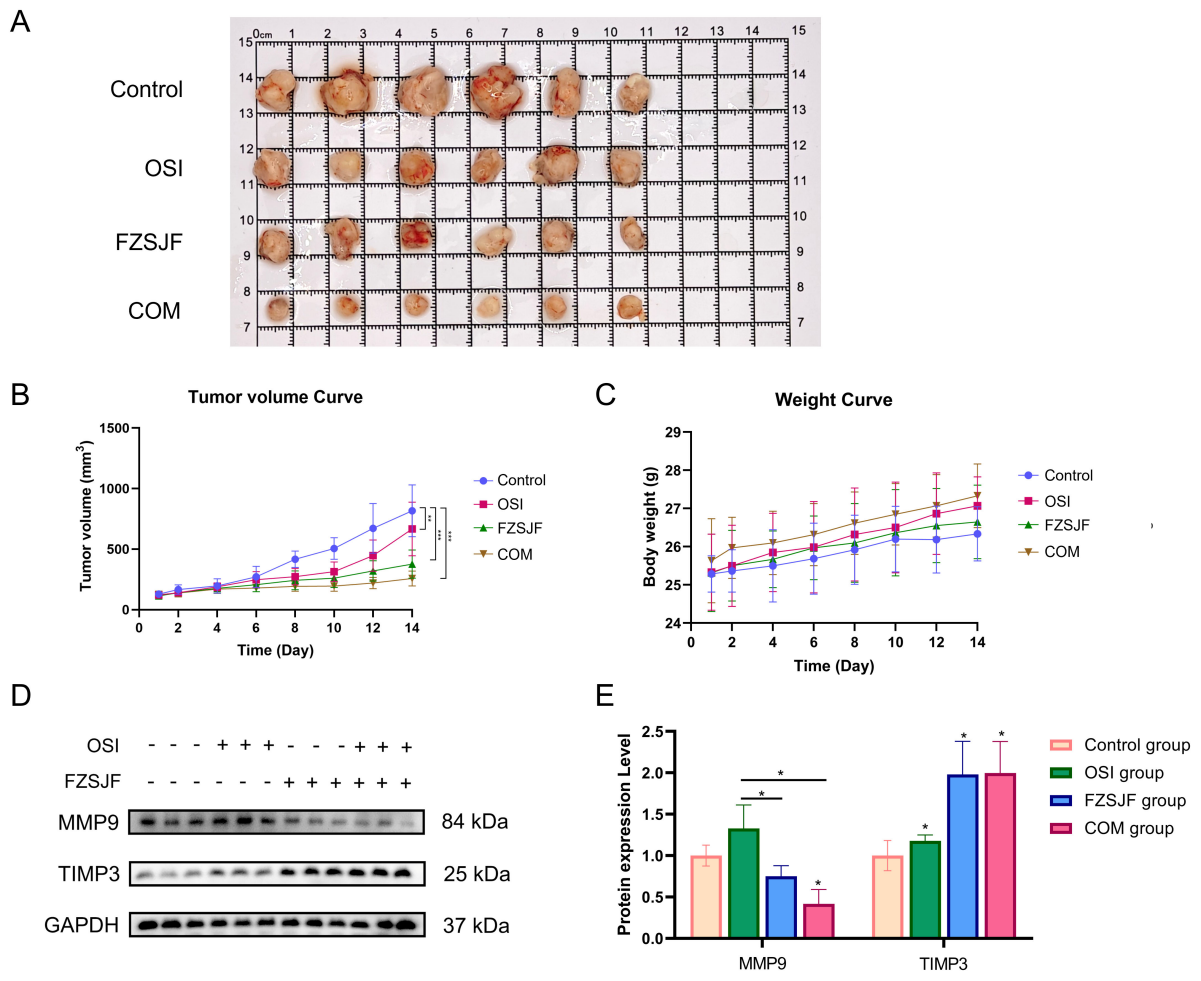
Intervention of the FZSJF combined with osimertinib in a nude mouse xenograft model of osimertinib-resistant NSCLC. **(A)** Excised tumors on day 14 (n=6); **(B)** Tumor volume growth curves (n=6); **(C)** Body weight change curves (n=6); **(D)** Representative WB bands (n=3); **(E)** Bar charts showing MMP9 and TIMP3 protein expression detected by WB (n=3). Error bars: SD. *P < 0.05, **P < 0.01, ***P < 0.001.

Nude mice were randomly divided into a control group, an osimertinib group, an FZSJF group, and a combination group, and received corresponding treatments. The results showed that, compared with the control group, tumor volumes in the osimertinib, FZSJF, and combination groups were significantly different, with the combination group exhibiting the best therapeutic effect (**Figures 16A, B**). Furthermore, no statistically significant differences in body weight were observed among the control, osimertinib, FZSJF, and combination groups (**Figure 15C**). These findings indicate that both the FZSJF group and the combination group can significantly delay the progression of osimertinib-resistant NSCLC.

##### 4.3.3.2 Effects on protein expression levels in tumor tissues

WB revealed that the FZSJF group and the combination group inhibited the expression of MMP9 (**Figures 16D, E**) and upregulated the expression of TIMP3 in the tumor tissues of nude mice (**Figures 16D, E**).

## 5 Discussion

The mortality rate of lung cancer has been increasing year by year, posing a serious threat to human health and life. Although significant progress has been made in surgery, radiotherapy, chemotherapy, targeted therapy, and immunotherapy, tumor metastasis and drug resistance remain challenging issues in clinical practice. As a unique healthcare resource in China, TCM has demonstrated distinct advantages in promoting “survival with tumor”, alleviating the toxic side effects of radiotherapy and chemotherapy, and preventing recurrence and metastasis in lung cancer patients. Numerous studies have shown that various active components of Chinese herbs and herbal formulas can effectively inhibit the migration and invasion of lung cancer cells and enhance the sensitivity to chemotherapeutic and targeted agents by interfering with relevant signaling pathways (17, 18).

In recent years, significant progress has been made in research on Fu-Zheng (healthy-qi reinforcing) Chinese herbal formulas for the treatment of NSCLC. Chen et al. systematically reviewed therapeutic strategies for combining Chinese herbal medicines with EGFR-TKIs to overcome NSCLC resistance, highlighting the advantages of multi-target modulation by herbal formulas (19). Jiang et al., using metabolomics and network pharmacology, demonstrated that FuZheng XiaoJi decoction exerts anti-NSCLC effects by inhibiting the PI3K/AKT and ERK/MAPK signaling pathways (20). In contrast to the above studies, the targets of FZSJF are not the classical tumor cell proliferation signaling pathways, but rather the homeostatic regulation of the CAFs-TIMP3-MMP9 axis within the tumor microenvironment. This finding provides a new perspective on the modern biological connotations of “Fu-Zheng” (reinforcing healthy qi) in Traditional Chinese Medicine—namely, indirectly regulating drug-resistant tumors by restoring protease-inhibitor homeostasis in the microenvironment, rather than directly killing tumor cells.

Lung cancer falls under the categories of *Fei Ji* (lung accumulation) and *Xi Ji* (breath accumulation) in Traditional Chinese Medicine (TCM). A deficiency of healthy Qi characterizes its pathogenesis (Zheng Xu) as the root cause, with phlegm, toxins, and blood stasis intermingling as the manifest branch symptoms. Internal injury-induced deficiency of Qi, blood, Yin, and Yang of the viscera constitutes the fundamental basis for the development of *Fei Ji*. Under this precondition, impaired transformation and transportation functions of the spleen and stomach lead to dysregulation of fluid distribution, resulting in the generation of phlegm turbidity (*Tan Zhuo*). Meanwhile, the inherent deficiency of healthy Qi fails to propel blood circulation, leading to blood stasis (*Yu Xue*). Phlegm turbidity and blood stasis interact, influencing each other and accumulating layer upon layer in a mutually reinforcing manner, eventually congealing into a mass and culminating in cancerous toxin (*Ai Du*). As the disease progresses in *Fei Ji* patients, pathological products arising from phlegm, stasis, and toxin progressively increase; the cancerous toxin repeatedly depletes healthy Qi, further weakening lung and spleen Qi. This creates a vicious cycle where insufficient healthy Qi allows pathogenic factors to flourish. At the same time, severe obstruction by phlegm and stasis ultimately renders the condition protracted and refractory, or even exacerbates the disease (21, 22). The development of osimertinib resistance in non-small cell lung cancer (NSCLC) can be interpreted in TCM theory as a dynamic pathological evolution of “medicinal toxicity depleting healthy Qi, leading to deep concealment of latent toxin and generation of novel pathogenic factors.” TCM categorizes this condition as “deficiency of healthy Qi with excess of pathogenic factors” (*Zheng Xu Xie Shi*). Although osimertinib can temporarily suppress cancerous toxins, its nature is inherently intense; continuous administration tends further to exhaust lung, spleen, and kidney Qi. Once healthy Qi is depleted, and the dynamic equilibrium between healthy Qi and pathogenic factors (*Zheng Xie*) is disrupted, the drug fails to exert its original efficacy. The residual cancerous toxin, deeply latent in the collateral vessels (Luo Mai), escapes regulation, intermingles with regenerated phlegm and stasis within the body, and eventually leads to the resurgence of pathogenic forces and drug failure, thereby providing opportunities for the cancerous toxin to re-aggregate and evolve.

Based on extensive clinical practice with osimertinib-resistant NSCLC patients, Professor Zhou Jihong from Guangzhou University of Chinese Medicine characterized these patients as manifesting “progressive weakening of lung Qi (*Fei Q*i) and chronic deficiency of spleen Qi (*Pi Qi)*,” and proposed the therapeutic principle of *“Fu Zheng San Jie*” (supporting healthy Qi and dissipating nodulation). Referencing the classical TCM formulary *Tai Ping Hui Min He Ji Ju Fang*, Professor Zhou modified the classical formula *Shen Ling Bai Zhu San* to create FZSJF, which achieves the effects of reinforcing healthy Qi, tonifying deficiency, reducing swelling, and resolving nodulation.

In FZSJF, *Hairy Fig Root* (*Wu Zhi Mao Tao*, *Fici Hirtae Radix*), sweet in flavor and neutral in nature, enters the spleen, lung, and liver meridians. It strengthens the spleen, tonifies the lung, promotes Qi movement, eliminates dampness, and relaxes the sinews and activates the collaterals, capable of supplementing Qi and tonifying deficiency without inducing warm-dryness. *Heterophylly Falsestarwort Root* (*Tai Zi Shen*, *Pseudostellariae Radix*), sweet and slightly bitter in flavor and neutral in nature, enters the spleen and lung meridians. It supplements Qi, strengthens the spleen, generates fluids, and moistens the lung, treating Qi deficiency by fortifying the spleen earth (*Pi Tu*), serving as a gentle tonifying agent for the lung and spleen. These two medicinals work synergistically to support healthy Qi, tonify deficiency, benefit the lung, and strengthen the spleen, together constituting the *Sovereign medicinals* (*Jun Yao*).

*Indian Bread* (*Fu Ling*, *Poria*), sweet and bland in flavor and neutral in nature, enters the heart, lung, spleen, and kidney meridians; *Largehead Atractylodes Rhizome* (*Bai Zhu*, *Atractylodis Macrocephalae Rhizoma*), sweet and bitter in flavor and slightly warm in nature, enters the spleen and stomach meridians. Both supplement Qi, strengthen the spleen, promote diuresis, and dry dampness, jointly assisting *Hairy Fig Root* and *Heterophylla Falsestarwort Root* in cultivating and replenishing healthy Qi and fortifying the spleen to tonify deficiency, together serving as the *Minister medicinals* (*Chen Yao*).

*Baical Skullcap Root* (*Huang Qin*, *Scutellariae Radix*), bitter in flavor and cold in nature, enters the lung, gallbladder, spleen, large intestine, and small intestine meridians, capable of clearing lung heat and dispelling pathogenic toxins; *Appendiculate Cremastra Pseudobulb* (*Shan Ci Gu*, *Cremastrae Pseudobulbus*), sweet and slightly pungent in flavor and cool in nature, enters the liver and spleen meridians, clearing heat, detoxifying, transforming phlegm, and dissipating nodulation; *Hedyotis Diffusa* (*Bai Hua She She Cao*, *Hedyotis Diffusae Herba*), bitter and bland in flavor and cold in nature, enters the stomach, large intestine, and small intestine meridians, capable of clearing heat, detoxifying, and reducing swelling; *Cat’s Claw Buttercup Root* (*Mao Zhao Cao*, *Ranunculi Ternati Radix*), sweet and pungent in flavor and warm in nature, enters the liver and lung meridians, capable of transforming phlegm turbidity and dissipating stagnation and nodulation; *Giant Knotweed Rhizome* (*Hu Zhang*, *Polygoni Cuspidati Rhizoma et Radix*), bitter in flavor and slightly cold in nature, enters the liver, gallbladder, and lung meridians, capable of resolving heat toxins and eliminating stasis swelling; *Zedoary* (*E Zhu*, *Curcumae Rhizoma*), pungent and bitter in flavor and warm in nature, enters the liver and spleen meridians, possessing the functions of breaking blood, moving Qi, dissipating stasis, and resolving accumulation; *Chinese Lobelia* (*Ban Bian Lian*, *Lobeliae Chinensis Herba*), pungent in flavor and neutral in nature, enters the heart, lung, and small intestine meridians, capable of activating blood, dissipating stasis, clearing heat, and detoxifying; *Oyster Shell* (*Mu Li*, *Ostreae Concha*), salty in flavor and slightly cold in nature, enters the liver, gallbladder, and kidney meridians, capable of softening hardness and resolving masses (*Xiao Zheng*). The aforementioned medicinals collectively achieve the effects of supporting healthy Qi, tonifying deficiency, dissipating nodulation, and reducing swelling, together serving as the *Assistant medicinals* (*Zuo Yao*).

*Platycodon Root* (*Jie Geng*, *Platycodonis Radix*), bitter and pungent in flavor and neutral in nature, enters the lung meridian, serving as the “carrier of all medicinals” (*Zhou Ji*), guiding the medicinal efficacy upward to enter the lung meridian, thus serving as the *Guide drug* (*Shi Yao*).

Through the harmonious integration of the seven emotional relationships (*Qi Qing*) among medicinals and their mutual compatibility, the formula collectively achieves the effects of supporting healthy Qi, tonifying deficiency, reducing swelling, and dissipating nodulation, and is indicated for treating *Fei Ji* (lung accumulation) of the lung-spleen Qi deficiency pattern (*Fei Pi Qi Xu Xing*).

In this study, a total of 124 active components of FZSJF were identified through comprehensive spectral component identification analysis, network pharmacology, and a review of the related literature. Meanwhile, KEGG pathway analysis revealed that FZSJF may influence NSCLC treatment by modulating pathways including “Pathways in cancer,” “Non-small cell lung cancer,” and “EGFR tyrosine kinase inhibitor resistance.” Among these, “Pathways in cancer” is a collection of cancer-related pathways that cover multiple core processes, such as cell proliferation, apoptosis, and angiogenesis, suggesting that FZSJF may intervene in the malignant biological behavior of tumors through multi-target mechanisms (23). Furthermore, the enrichment of the “Non-small cell lung cancer” pathway directly points to the regulatory effects of this formula on NSCLC-specific driver genes and their downstream signaling networks. EGFR-TKIs (e.g., gefitinib and osimertinib) are the standard treatment for EGFR-mutant NSCLC (24), and the significant enrichment of FZSJF in the “EGFR tyrosine kinase inhibitor resistance” pathway indicates its potential value in reversing EGFR-TKI resistance. In addition, molecular docking results demonstrated that MMP9 and TIMP3 exhibited very high docking scores with the main active components of FZSJF as well as with osimertinib.

In the animal experiment of this study, tumor growth in the H1975N+CAFs and H1975OR groups was significantly faster than in the H1975N group. Moreover, there was a statistically significant difference in body weight between the two groups, with the H1975N+CAFs group showing significantly lower body weight than the H1975N group. The tumor microenvironment (TME) refers to the internal and external environment in which tumor cells arise, develop, and metastasize, including the tissue, structure, and metabolic state surrounding them. CAFs are the most abundant stromal cell type in the NSCLC tumor microenvironment. CAFs participate in tumor progression and ultimately affect therapeutic outcomes through multiple mechanisms, including paracrinally secreting cytokines, remodeling extracellular matrix (ECM) components, influencing the immune microenvironment, and regulating the recruitment or polarization of other cell populations. Consequently, CAFs have been termed the “conductors and coordinators” of tumors (25, 26). The high heterogeneity and plasticity of CAFs enable them to play critical roles in tumor initiation, progression, and drug resistance. Therefore, their functions and underlying mechanisms in tumor development have gradually gained increasing attention and research interest in recent years (27).

Data mining of the TCGA database in this study revealed that MMP9 was upregulated and TIMP3 was downregulated in tumor tissues. Paired analysis further demonstrated significant differences in MMP9 and TIMP3 expression between normal and tumor tissues. Spearman’s analysis showed a significant correlation between MMP9 and TIMP3 expression levels. Kaplan–Meier curve analysis was performed to evaluate the impact of MMP9 and TIMP3 expression levels on OS of lung cancer patients. The results showed that lung cancer patients with high MMP9 expression had significantly shorter OS than those with low MMP9 expression. In contrast, patients with high TIMP3 expression had significantly longer OS than those with low TIMP3 expression. The prognostic value of MMP9 and TIMP3 observed in our TCGA data mining aligns with earlier clinical studies demonstrating that high MMP9 expression correlates with poor prognosis and unfavorable clinicopathological characteristics in NSCLC (28, 29). At the same time, TIMP3 deficiency is associated with worse outcomes (30).

In the cellular experiments of this study, H1975OR cells exhibited higher MMP9 protein and mRNA expression levels and lower TIMP3 expression levels compared with H1975N cells. Similarly, compared with NAFs, CAFs showed higher MMP9 protein and mRNA expression levels and lower TIMP3 expression levels. Regarding drug treatment, this study demonstrated that FZSJF alone or in combination with osimertinib effectively inhibited the proliferation, invasion, and migration of H1975OR cells. Moreover, the cellular experiments showed that FZSJF, alone or in combination with osimertinib, effectively upregulated TIMP3 expression and downregulated MMP9 expression in both H1975OR cells and CAFs. In the animal experiments, the results revealed that, compared with the H1975N group, the tumor tissues of nude mice in the H1975OR group and the H1975N+CAFs group exhibited higher MMP9 expression and lower TIMP3 expression. Administration of FZSJF alone or in combination with osimertinib effectively reduced the tumor growth rate in nude mice, upregulated TIMP3 expression, and downregulated MMP9 expression in tumor tissues.

Our finding that CAFs exhibit elevated MMP9 expression and reduced TIMP3 expression is consistent with previous reports demonstrating that CAFs are a major source of MMP9 in the tumor microenvironment (31–33). That loss of TIMP3 promotes CAF activation and ECM remodeling (34). MMP9, also known as gelatinase B, is a zinc-dependent and calcium-regulated endoproteinase belonging to the matrix metalloproteinase family. It mediates basement membrane and matrix remodeling by degrading ECM components, including type IV collagen and gelatin (35). Under physiological conditions, MMP9 participates in tissue development, inflammatory responses, and wound repair (7). During tumor initiation and progression, high MMP9 expression is closely associated with tumor cell invasion, metastasis, and angiogenesis. MMP9 can contribute to drug resistance through multiple direct and indirect mechanisms. On one hand, MMP9 degrades major components of the ECM and basement membrane, thereby weakening the physical barrier between the tumor and surrounding tissues. This creates conditions for tumor cells to break through the primary site, achieve infiltration, and undergo distant metastasis. Consequently, even if osimertinib inhibits some cells at the primary site, this enhanced invasiveness can still lead to treatment failure and disease progression (35). On the other hand, MMP9 can release or activate latent bioactive factors (such as TGF-β and VEGF), promoting EMT in tumor cells and further enhancing their migration and invasive capacity (36). Therefore, MMP9 is considered to play a critical role in malignant tumor progression and to serve as a key effector molecule linking CAFs to osimertinib resistance, with potential value as a prognostic predictor and therapeutic target.

TIMPs are broad-spectrum and potent endogenous inhibitors of the MMP family. They can directly block the proteolytic activity of MMP9 by binding to its catalytic zinc ion site (8). TIMP3 is a member of the TIMP family and is a secreted glycoprotein encoded on chromosome 22q12.1-q13.2. Its mechanisms of action include regulating cell death, angiogenesis, tumor inflammation, and tumor cell invasion and dissemination, thereby playing an important role in tumor progression (11, 37). Impaired expression or function of TIMP3 may lead to a relative excess of MMP9 activity, enhanced ECM degradation, reduced drug penetration, and positive-feedback maintenance of CAF activation. Multiple studies have shown that reduced or absent TIMP3 expression is associated with poor prognosis, including increased tumor size, higher tumor stage, and metastasis, compared with normal control groups (38). Absolute or relative changes in the levels of MMPs or TIMPs can induce significant alterations in the activity of specific MMPs, and the MMPs/TIMPs ratio often determines the extent of extracellular matrix protein degradation and tissue remodeling (39).

It should be noted that the present study also has certain limitations. First, although no significant body-weight loss was observed during treatment, this result should be interpreted only as preliminary evidence of treatment tolerability. Body weight alone is insufficient to fully establish systemic safety. Because the formula contains multiple herbal components, including compounds with reported potential hepatotoxicity or nephrotoxicity, further histopathological examination of the liver and kidney and serum biochemical analyses such as ALT, AST, and creatinine are required in future studies to comprehensively evaluate its safety profile. Consequently, a comprehensive toxicity profile of FZSJF, particularly its long-term or off-target effects, could not be fully assessed. Second, this study investigated only the overall effects of the FZSJF formula on osimertinib-resistant NSCLC; the regulatory mechanisms of the individual active components of FZSJF in this context remain to be elucidated further. Finally, it should be noted that the TGF-β-induced HFL1 model mainly reflects a myofibroblastic CAF-like phenotype and does not fully recapitulate the heterogeneity of CAF populations in patient tumors. *In vivo* tumors contain multiple CAF subtypes with distinct molecular features and biological functions, including inflammatory, antigen-presenting, and other context-dependent CAF states. Therefore, the present findings should be interpreted primarily in the context of TGF-β-driven myofibroblastic CAF activation. Future studies using primary patient-derived CAFs, multiple CAF subtypes, or single-cell-based validation will be needed to determine whether these findings can be generalized to broader CAF populations.

## 6 Conclusion

In summary, the results of network pharmacology, molecular docking, data mining, cellular experiments, and animal experiments in this study collectively indicate that CAFs, TIMP3, and MMP9 constitute a fine and dynamic regulatory network. CAFs can secrete MMP9, and high MMP9 expression may contribute to osimertinib resistance in NSCLC. Conversely, high TIMP3 expression may reduce MMP9 expression, thereby reversing osimertinib resistance in NSCLC. Furthermore, this study suggests that the mechanism by which FZSJF reverses NSCLC resistance may involve increasing TIMP3 expression and, consequently, inhibiting MMP9 expression, providing fundamental experimental evidence and a basis for the use of FZSJF in the treatment of osimertinib-resistant NSCLC.

## Data Availability

The original contributions presented in the study are included in the article/supplementary material. Further inquiries can be directed to the corresponding author.
